# AMP-activated protein kinase mediates adaptation of glioblastoma cells to conditions of the tumor microenvironment

**DOI:** 10.1186/s13046-025-03346-2

**Published:** 2025-03-24

**Authors:** Nadja I. Lorenz, Benedikt Sauer, Hans Urban, Jan-Béla Weinem, Bhavesh S. Parmar, Pia S. Zeiner, Maja I. Strecker, Dorothea Schulte, Michel Mittelbronn, Tijna Alekseeva, Lisa Sevenich, Patrick N. Harter, Christian Münch, Joachim P. Steinbach, Anna-Luisa Luger, Dieter Henrik Heiland, Michael W. Ronellenfitsch

**Affiliations:** 1 Dr. Senckenberg Institute of Neurooncology, University Hospital Frankfurt, Goethe University, Frankfurt am Main, Germany; 2 German Cancer Consortium (DKTK), Partner Site Frankfurt/Mainz, Frankfurt am Main, Germany; 3 Frankfurt Cancer Institute (FCI), University Hospital Frankfurt, Goethe University, Frankfurt am Main, Germany; 4 University Cancer Center Frankfurt (UCT), University Hospital Frankfurt, Goethe University, Frankfurt am Main, Germany; 5 Institute of Molecular Systems Medicine, Goethe University, Frankfurt am Main, Germany; 6 Institute of Neurology (Edinger Institute), University Hospital Frankfurt, Goethe University, Frankfurt am Main, Germany; 7 Luxembourg Centre of Neuropathology (LCNP), Dudelange, Luxembourg; 8 Luxembourg Centre for Systems Biomedicine (LCSB), University of Luxembourg, Esch-sur-Alzette, Luxembourg; 9 National Center of Pathology (NCP), Laboratoire National de Santé (LNS), Dudelange, Luxembourg; 10 Department of Cancer Research (DoCR), Luxembourg Institute of Health (LIH), Strassen, Luxembourg; 11 Faculty of Science, Technology and Medicine (FSTM), University of Luxembourg, Esch-sur- Alzette, Luxembourg; 12 Department of Life Science and Medicine (DLSM), University of Luxembourg, Esch-sur- Alzette, Luxembourg; 13 Institute for Tumor Biology and Experimental Therapy, Georg-Speyer-Haus, Frankfurt am Main, Germany; 14 Center for Neuropathology and Prion Research, Faculty of Medicine, Ludwig-Maximilians- University of Munich, Munich, Germany; 15 Cardio-Pulmonary Institute, Frankfurt am Main, Germany; 16 Microenvironment and Immunology Research Laboratory, Medical Center, University of Freiburg, Freiburg, Germany; 17 Department of Neurosurgery, Medical Center, University of Freiburg, Freiburg, Germany; 18 Faculty of Medicine, University of Freiburg, Freiburg, Germany; 19 Comprehensive Cancer Center Freiburg (CCCF), Faculty of Medicine and Medical Center, University of Freiburg, Freiburg, Germany; 20 German Cancer Consortium (DKTK), Partner site Freiburg, Freiburg, Germany; 21 Department of Neurosurgery, University Clinic, Friedrich-Alexander University Erlangen-Nürnberg, Erlangen, Germany; 22 Department of Neurology, University Hospital Frankfurt, Goethe University, Frankfurt am Main, Germany

**Keywords:** Glioblastoma, AMP-activated protein kinase, AMPK, Metabolic adaptation, Hypoxia

## Abstract

AMP-activated protein kinase (AMPK) is an energy sensor that regulates cellular metabolic activity. We hypothesized that in glioblastoma (GB), AMPK plays a pivotal role in balancing metabolism under conditions of the tumor microenvironment with fluctuating and often low nutrient and oxygen availability. Impairment of this network could thus interfere with tumor progression. AMPK activity was modulated genetically by CRISPR/Cas9-based double knockout (DKO) of the catalytic α1 and α2 subunits in human GB cells and effects were confirmed by pharmacological AMPK inhibition using BAY3827 and an inactive control compound in primary GB cell cultures. We found that metabolic adaptation of GB cells under energy stress conditions (hypoxia, glucose deprivation) was dependent on AMPK and accordingly that AMPK DKO cells were more vulnerable to glucose deprivation or inhibition of glycolysis and sensitized to hypoxia-induced cell death. This effect was rescued by reexpression of the AMPK α2 subunit. Similar results were observed using the selective pharmacological AMPK inhibitor BAY3827. Mitochondrial biogenesis was regulated AMPK-dependently with a reduced mitochondrial mass and mitochondrial membrane potential in AMPK DKO GB cells. In vivo, AMPK DKO GB cells showed impaired tumor growth and tumor formation in CAM assays as well as in an orthotopic glioma mouse model. Our study highlights the importance of AMPK for GB cell adaptation towards energy depletion and emphasizes the role of AMPK for tumor formation in vivo. Moreover, we identified mitochondria as central downstream effectors of AMPK signaling. The development of AMPK inhibitors could open opportunities for the treatment of hypoxic tumors.

**Supplementary Information:**

The online version contains supplementary material available at 10.1186/s13046-025-03346-2.

## Background

Glioblastoma (GB) is a heterogeneous diffuse glial tumor with distinct histological features including necrosis and neoangiogenesis that mirror fluctuating nutrient deprivation and hypoxia in the tumor microenvironment, potential drivers of progression of solid tumors [1]. Standard GB therapy comprises surgery followed by radiotherapy and alkylating chemotherapy with temozolomide [2]. Recent clinical development has failed to identify additional drugs for the limited arsenal of GB treatment options, thus novel therapeutic strategies are urgently needed.

Altered metabolism has been recognized as a hallmark of cancer and might expose a targetable Achilles’ heel [3]. Glucose is considered the major energy source for cancer cells and oxidation *via* glycolysis and subsequent oxidative metabolism generates energy by increasing adenosine triphosphate (ATP) levels. While human physiology aims at maintaining a steady state of serum nutrients and oxygen, cells are additionally equipped with an intrinsic machinery to adjust metabolism based on energy supply [4]. At the core of this mechanism is 5′-adenosine monophosphate (AMP)-activated protein kinase (AMPK) which is activated by an increase of the AMP/ATP ratio [5] as well as glucose deprivation *via* the glycolytic enzyme aldolase [6, 7] and orchestrates adaptation of metabolism during nutrient starvation to promote cell survival. The heterotrimeric protein AMPK is composed of a catalytic α subunit that contains the phosphorylation site Thr172 important for its activation and the regulatory β and γ subunits [8]. Notably, several isoforms of these proteins encoded by distinct genes exist in mammalian cells. During energy stress conditions AMP or ADP binding to the γ subunit of AMPK triggers phosphorylation of Thr172 of the α subunit by upstream kinases, mostly by the liver kinase B1 (LKB1) [9]. To maintain energy homeostasis in states of low external energy supply, AMPK directs metabolism towards catabolism e.g. by activating fatty acid oxidation and glycolysis for ATP production [5]. AMPK phosphorylation targets include acetyl-CoA carboxylase (ACC), which catalyzes the first step of fatty acid synthesis and is inhibited by Ser79 phosphorylation. This phosphorylation is frequently used as a surrogate marker for AMPK activity [10, 11]. *Via* phosphorylation of its targets tuberin (TSC2) and Raptor, AMPK also indirectly inhibits mammalian target of rapamycin complex 1 (mTORC1) signaling which integrates signals from growth factor receptors with the metabolic state of the cell to regulate diverse targets involved in translation, cell growth and autophagy [12].

Besides its role in metabolic programming to counteract starvation conditions, AMPK regulates mitochondrial dynamics including fusion and fission states and homeostasis as well as mitochondrial quality control by regulation of mitophagy [13, 14]. AMPK has also been implicated in the regulation of energy homeostasis via peroxisome proliferator-activated receptor gamma coactivator 1-alpha (PGC-1α) in MEFs and H1299-EV cells [15].

It therefore is plausible that AMPK exerts important functions for cellular stress adaptation in tumors. However, there are conflicting results with regard to pro- and anti-tumor effects for different types and stages of cancer [16–18]. The fact that lung cancers frequently display LKB1 mutations with potentially reduced levels of AMPK activity [19] indicates a tumor suppressive role of AMPK. In contrast, recently, a chronic activation of AMPK by oncogenic stress in GB cells has been reported to regulate hypoxia-inducible factor 1α and glycolysis *via* phosphorylation of the transcription factor CREB1 to enhance tumor growth and GB bioenergetics [18]. In this context, inhibition of AMPK by gene suppression of the β1 regulatory subunit led to reduced tumor growth in vitro and in vivo together with reduced glycolysis and oxidative phosphorylation [18]. So far, available pharmacologic AMPK inhibitors lack specificity and hence are not well-suited for evaluation of AMPK as a therapeutic target in preclinical studies and clinical studies. For example, one of the most commonly employed AMPK inhibitors, Compound C, interferes with several other kinases leading to AMPK-independent effects [20–22].

In this experimental study, we investigated the role of the AMPK-mediated stress response in human GB cells. Mimicking conditions of the tumor microenvironment we deprived GB cells of glucose and oxygen to characterize metabolic effects and to delineate the relevance of potential downstream targets. AMPK inhibition was modelled by using double-knockout cells of the catalytic subunits α1 and α2. We further demonstrated the specificity of a novel pharmacological AMPK inhibitor which was used for validation experiments in primary GB cell cultures. We here report that AMPK activation promotes GB cell survival in the context of both hypoxia and glucose starvation by promoting metabolic adaptation. Furthermore, knockout of the catalytic AMPK subunits impaired tumor formation and prolonged survival in a mouse model. Taken together, the results of our study identify AMPK as a therapeutic target for inhibition in GB.

## Methods

### Reagents, cell lines and culture conditions

LNT-229 cells have been described [23]. LN-308 cells were a gift of Nicolas de Tribolet (Lausanne, Switzerland); G55T2 cells were provided by Manfred Westphal and Kathrin Lamszus (Hamburg, Germany) [24]. All cell lines were maintained in cell culture incubators at 37° C under a 5% CO_2_ atmosphere. Cells were cultured in Dulbecco’s modified eagle medium (DMEM) containing 10% fetal calf serum (FCS) (Thermo Fisher Scientific, Hamburg, Germany), 100 IU/ml penicillin and 100 µg/ml streptomycin (Life Technologies, Karlsruhe, Germany) [23].

LNT-229 and LN-308 cells were authenticated by STR analysis (Multiplexion, Heidelberg, Germany). The STR profile of LNT-229 cells matched with the known profile for LN-229, which only differ in their p53 status [25]. A STR profile of G55T2 cells has not been deposited in databases yet [26].

All reagents, if not specified elsewhere, were purchased from Sigma (Taufkirchen, Germany). BAY974 and BAY3827 were kindly provided by the DCP (Donated Chemical Probes) program [27].

### Generation of CRISPR/Cas9 knockout cells

For CRISPR/Cas9 knockout AMPK sgRNA plasmids targeting exon 1 of AMPK α1 and α2 (pX462-hPRKAA1-gRNA, pX462-hPRKAA2-gRNA, #74374–74377) were purchased from Addgene (Watertown, MA, USA). LNT-229, G55T2 and LN-308 cells were transfected with a combination of the gRNA plasmids (0.625 µg each) using Lipofectamine 3000 (Thermo Fisher Scientific, Hamburg, Germany) for 6 h. After 3 days, cells were selected with puromycin (2 µg/ml). Single cell clones were analyzed for AMPK expression by immunoblotting.

### Human primary GB cell culture

P3NS cells were a gift of Simone Niclou (Luxembourg Institute of Health, Luxembourg) and were cultured in Neurobasal A medium supplemented with 1x B27 supplement, 2% glutamine, 1 U/ml heparin, 20 ng/ml epidermal growth factor (EGF) and 20 ng/ml human recombinant basic fibroblast growth factor (bFGF) (ReliaTech, Wolfenbüttel, Germany) [28]. NCH690 and NCH644 cells were purchased from CLS (Eppelheim, Germany) and were cultured in Neurobasal A medium (Thermo Fisher Scientific, Dreieich, Germany) supplemented with 1x B27 supplement (Thermo Fisher Scientific), 2 mM glutamine (Thermo Fisher Scientific), 1 U/ml heparin, 100 IU/ml penicillin and 100 µg/ml streptomycin (Life Technologies, Karlsruhe, Germany) and 20 ng/ml EGF and FGF-2 (ReliaTech).

### Transfection of cells

For expression of wildtype AMPK α2 (*PRKAA2*) in LNT-229 AMPK α1/2 double knockout cells, pcDNA3.1 plasmids with the according constructs were purchased from GenScript (Leiden, The Netherlands). Transfection with Attractene (Qiagen, Venlo, Netherlands) was performed according to the manufacturer’s protocol and the empty pcDNA3.1 plasmid was used as control. For selection, medium containing 400 µg/ml G418 was used. The efficacy of transfection was checked in early passage pooled clones.

### Induction of hypoxia

Hypoxia was induced with incubation in GasPak pouches for anaerobic culture (Becton-Dickinson, Heidelberg, Germany) as previously described [23, 26, 29]. Experiments in hypoxia (0.1% O_2_) or normoxia (21% O_2_) were performed in serum-free DMEM adjusted to 2 mM glucose.

### Cell density and viability assays

Cell density was assessed by crystal violet (CV) staining as previously described [30]. For cell viability measurements, propidium iodide (PI) uptake was quantified by flow cytometry (FACS) as previously described [23]. A BD Canto II flow cytometer was used for data acquisition and data analysis was performed with the BD FACS Diva software version 6.1.3. Cell viability analysis by lactate dehydrogenase (LDH) release assay was performed with the Cytotoxicity Detection Kit (LDH) (Roche, Mannheim, Germany) and has also been described [23, 31].

### Immunoblot analysis

Immunoblot analyses were performed following a standard protocol [32] with the following antibodies: pACC (Ser79), pAMPK (Thr172), ACC and AMPKα1/2 (#3661, #2535, #3661, #2532, Cell Signaling Technology, Danvers, MA, USA), AMPKα2 (#18167-1, Proteintech, Rosemont, IL, USA) and actin (#sc-1616, Santa Cruz Biotechnology, Santa Cruz, CA, USA). Secondary anti-rabbit and anti-goat antibodies were purchased from Jackson ImmunoResearch (#111-036-144; West Grove, PA, USA) and Santa Cruz Biotechnology (#sc-2020).

### RNA isolation and quantitative real-time PCR

RNA extraction and cDNA synthesis was performed as described [32]. Absolute SYBR Green Fluorescein q-PCR Mastermix (Thermo Fisher Scientific) was used for quantitative RT-PCR measurements with the corresponding primers, *18 S* and *SDHA* were used as housekeeping genes for normalization [32].

### Measurement of oxygen consumption

Cells were seeded and incubated overnight to ensure cell attachment. Cultured cells were treated as indicated and overlaid with sterile paraffin oil. Oxygen consumption was determined with a fluorescence-based assay (PreSens, Regensburg, Germany) [32].

### Determination of mitochondrial mass and mitochondrial membrane potential by flow cytometry

Changes in mitochondrial mass and mitochondrial membrane potential were measured by flow cytometry. Briefly, cells were seeded and allowed to attach overnight. Cells were treated as indicated. After washing with PBS, cells were stained with 100 nM MitoTracker Green FM or Mitotracker Red (Thermo Fisher Scientific, Hamburg, Germany) for 20 min. Afterwards cells were harvested, washed and analyzed by flow cytometry (BD Canto II).

### Chorioallantoic membrane (CAM) assay

Fertilized chicken eggs (LSL Rhein-Main, Dieburg, Germany) were incubated at 37° C for seven days after fertilization. 2 × 10^6^ cells were diluted in 10 µl DMEM and 10 µl Corning Matrigel (Corning, Amsterdam, Netherlands) and placed onto a blood vessel of the chorioallantoic membrane. Tumors were isolated after seven further days of incubation. Tumor weight was documented and tumors were analyzed by immunohistological staining.

### Immunohistochemistry

Immunohistochemistry (IHC) analyses were performed with formalin-fixed paraffin-embedded (FFPE) tissue of mouse and CAM tumors. The following antibodies were used: anti-CA9 (#5649, clone D47G3, dilution 1:100, Cell Signaling Technology, Danvers, MA, USA), anti-p-ACC (Ser79) (#3661, dilution 1:200, Cell Signaling Technology, Danvers, MA, USA) and anti-p-AMPK (Thr172) (#2535, dilution 1:100, Cell Signaling Technology, Danvers, MA, USA). Tissue blocks were cut in slices of 3 μm thickness using a microtome (Leica Microsystems GmbH, Nussloch, Germany) and placed onto SuperFrost slides (Thermo Scientific). IHC was performed according to standardized protocols using Leica Bond RX automated immunostaining system (Leica Microsystems). IHC stainings were analyzed using a light microscope (BX41, Olympus, Hamburg, Germany).

### Mass spectrometry

For proteomic analysis, cells were seeded in triplicates. Further processing steps have previously been described [33]. Additional details regarding sample preparation, fractionation and liquid chromatography mass spectrometry have been included in the supplementary methods.

### Protein network analysis

Weighted Correlation Network Analysis (WGCNA) [34, 35] was used for protein co-correlation network analysis of mass spectrometry data leveraging the wildtype control and AMPK double knockout (DKO) of both catalytic subunits α1 and α2 as trait features to identify group specific protein modules. To initiate the model, the optimal soft-power for achieving scale-free topology (SFT) was determined by fitting a regression model with different soft-power thresholds p = {1,…,20} which identified *p* = 16 to reach SFT. To assemble a signed co-expression network, the topological overlap matrix (TOM) of the hdWGCNA::ConstructNetwork function was employed. Visualization of the co-expression network was done with Uniform Manifold Approximation and Projection for dimension reduction (ModuleUMAPPlot). Quantification of hub genes of each of the modules was achieved by calculating module connectivity (ModuleConnectivity). Then, using the top 100 module-associated genes (compareCluster), a gene ontology analysis was performed. The clusterProfiler package (dotplot) [36] was used for visualization of the pathways related to the identified modules and, finally, the correlation between each co-expression module and the cell line AMPK status was computed.

### In vivo experiments

All in vivo mouse experiments were approved by the local animal ethics committee (regional board Darmstadt, Germany), four-week-old female athymic nude mice (Crl: NU (NCr) Foxn1^nu^) were purchased from Charles River (Sulzfeld, Germany). Group sizes were determined based on the expected effect size for each cell line. For tumors derived from G55T2 cells, a larger effect size (Cohen’s d ≈ 0.8), based on previous data, was projected, allowing sufficient statistical power with 10 mice per group. In contrast, tumors derived from LNT-229 cells were expected to exhibit a smaller effect size (Cohen’s d < 0.5), necessitating 20 mice per group to maintain equivalent power. This approach ensured that both cell lines were assessed with adequate sensitivity to detect meaningful treatment effects despite anticipated differences in response magnitude. Animals were fed with standard food and water ad libitum and housed on a 12 h dark/night cycle in the local animal facility. Mice were allowed to acclimate in the local animal facility before start of the experiment. Mice from different cages were picked at random, anesthetized and received metamizol for pain relief. For tumor cell injection, mice were placed into a stereotactic fixation device and 1 × 10^4^ G55T2 wt or AMPK DKO cells or 1 × 10^5^ LNT-229 wt or AMPK DKO cells resuspended in 2 µl PBS were injected into the right striatum through a burr hole in the skull using a 10 µl Hamilton syringe. Wounds were closed with Surgibond tissue adhesive. After tumor cell injection, all animals were checked twice daily and sacrificed when neurological symptoms or a loss of more than 20% of body weight were determined. Brains were isolated and fixed in 4% PFA for immunohistochemical analysis. The mouse groups (wt vs. AMPK DKO) were not blinded to the experimenter.

Tumor growth was monitored by MRI. MRI measurements were performed at the Animal Imaging Core Facility after 11, 18 and 25 days using a 7 Tesla small animal MRI (Pharmascan, Bruker) and analyzed using ITK Snap software [37]. No mice were excluded from the analysis.

### Statistical analysis

All quantitative data are expressed as mean and standard deviation (SD). P-values were calculated with two-tailed student’s t-tests (Excel, Microsoft, Seattle, WA, USA). Values of *p* > 0.05 were considered as not significant (n.s.), values of *p* < 0.05 and *p* < 0.01 as significant or highly significant. For Kaplan-Meier analysis in in vivo experiments GraphPad Prism software was used.

### Data Availability

The mass spectrometry dataset has been deposited at the PRIDE-Proteomics Identification Database (submission ID PXD055976). All other datasets used or analyzed during the current study are available from the corresponding author upon reasonable request.

## Results

### AMPK pathway activation is a characteristic trait of GB

**Fig. 1:**
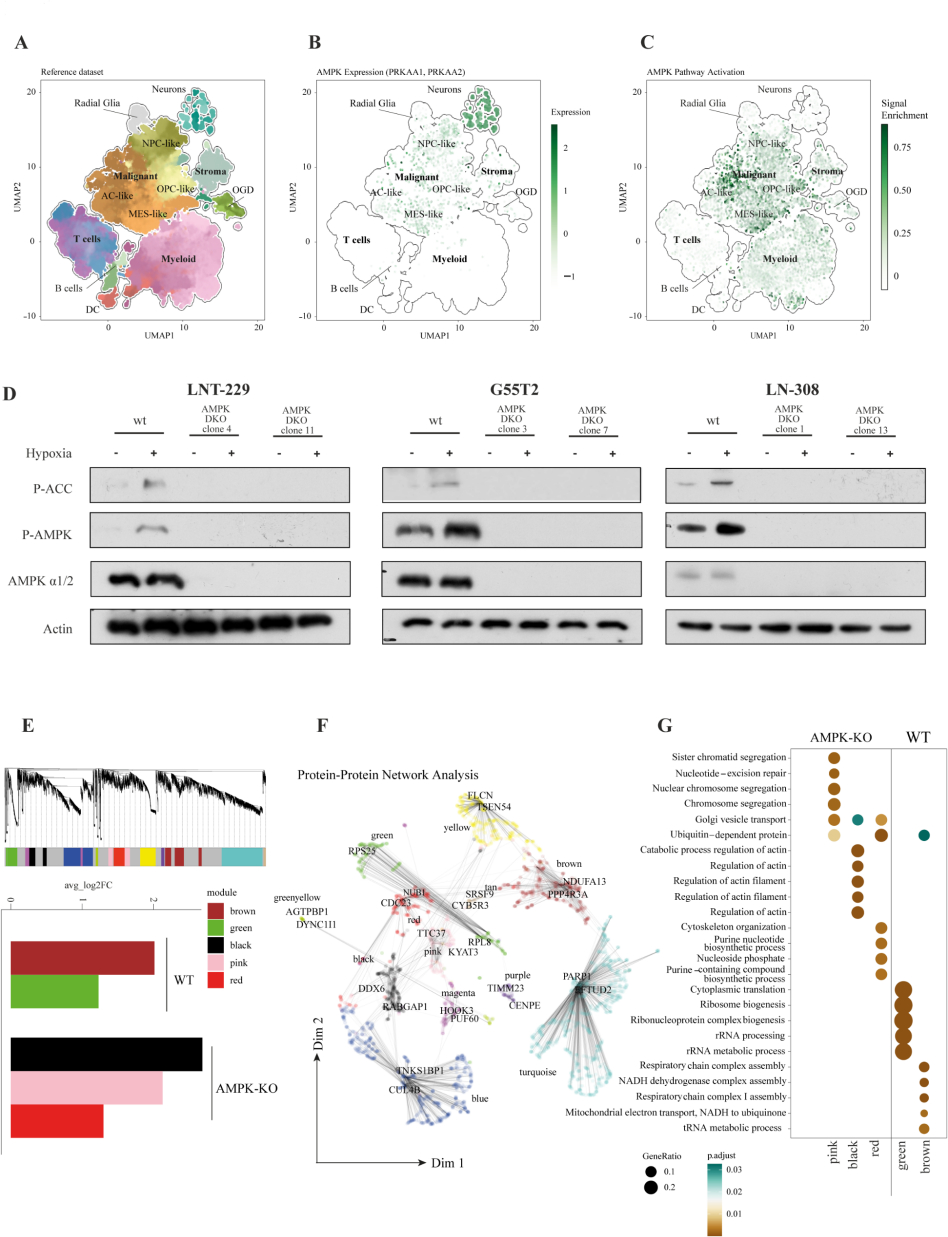
Knockout of AMPK catalytic subunits prevents AMPK activation and deregulates metabolic adaptation under hypoxic conditions in human GB cells (**A**) Dimensional reduction (UMAP) of the single cell reference GBMap dataset including neurons from the Allen Institute database. Celltypes are colored based on their transcriptional subtypes. (**B**, **C**) UMAP representation of AMPK gene expression (**B**) and for the AMPK pathway activation (**C**). (**D**) LNT-229, LN-308 and G55T2 wildtype (wt) or AMPK catalytic subunits double knockout (DKO) cells were incubated in serum-free DMEM supplemented with 2 mM glucose for 8 h in normoxia (21% O_2_) or hypoxia (0.1% O_2_). Immunoblots with antibodies for P-ACC, P-AMPK, AMPK α1/2 were performed. (**E**) Protein network analysis of LNT-229 wildtype and AMPK DKO cells using WGCNA. Representation of the modules is indicated in the Dendrogram in the upper panel. The differentially enriched modules across the conditions are demonstrated in the bottom panel. (**F**) UMAP representation of the protein expression modules. (**G**) Geneset Enrichment analysis of the modules which indicate significance between LNT-229 wildtype and AMPK DKO

To elucidate patterns of AMPK signaling, we analyzed the different cell types of the GB tumor microenvironment for expression and pathway activation of AMPK in the publicly available GBMap dataset. GBMap comprises multiple scRNA-seq datasets and thereby creates a cellular map of GB, which is a valuable resource for exploratory analysis, hypothesis generation and testing [38, 39]. We used the annotation-level 4 which includes the most detailed annotation of different GB subtypes and environmental cells (Fig. 1A). Our analyses revealed a prominent upregulation of AMPK expression among all malignant subgroups of GB. Interestingly, we also found high levels of AMPK in neurons (Fig. 1B). We next checked the activation of the pathway downstream of AMPK based on a select number of genes (Supplementary Table 1) and found markedly increased activity within the tumor cell population compared to the other microenvironmental cell populations. Some pathway activity was also detectable in myeloid cells, while in neurons (despite relatively high expression levels) no significant activity was detectable (Fig. 1C). This does not come unexpected because AMPK is activated during metabolic stress to which neurons are rarely exposed to.

### Double knockout of both catalytic AMPK subunits inhibits signaling under energy stress conditions in human GB cells

AMPK is known as a central cellular energy sensor that is necessary to switch to an increased catabolism during cellular energy stress conditions [8]. To elucidate the effect of AMPK activity for GB cells, LNT-229, LN-308 and G55T2 cells with a double knockout (DKO) of the catalytic subunits α1 and α2 were generated by CRISPR/Cas9 gene editing. While other studies had used GB cells with a knockdown of the regulatory AMPK β subunit [18], AMPK α1/α2 DKO cells offer the benefit of lacking catalytic activity and are therefore important tools to investigate AMPK-dependent effects in GB cells. LNT-229, G55T2 and LN-308 DKO cells showed no AMPK specific band in immunoblot analysis (Supplementary Fig. 1A). Two clones of each cell line were used for further experiments. Concomitant glucose and oxygen starvation, mimicking the conditions of the GB microenvironment with low oxygen and nutrient availability, resulted in an AMPK-mediated phosphorylation of ACC as a surrogate marker for AMPK activity, whereas ACC phosphorylation was absent in all tested AMPK DKO GB cell lines (Fig. 1D).

### Proteomic analysis of AMPK DKO cells reveals decreased abundance of proteins related to mitochondrial metabolism

We wondered whether the knockout of the AMPK catalytic subunit genes impacted the proteomic landscape in our GB cell models. To this end, we analyzed the proteome of LNT-229 AMPK DKO cells using a network-based approach known as Weighted Gene Co-expression Network Analysis (WGCNA). This method allowed us to correlate cell status (wildtype/DKO) with regulatory modules, thereby reducing potential confounding effects and enhancing our understanding of the underlying biological processes. Our correlation analysis between cell condition (wildtype/DKO) and protein modules revealed a significant reduction in the brown and green modules in LNT-229 AMPK DKO cells compared to LNT-229 wildtype cells, along with an enrichment of the black, pink, and red modules (Fig. 1E). The protein-protein network identified is visualized using Uniform Manifold Approximation and Projection (UMAP) (Fig. 1F). Subsequently, we performed gene set enrichment analysis on these modules to explore the biological functions upregulated (pink, black, and red) and downregulated (brown and green) in response to AMPK knockout (Fig. 1G). Notably, the brown module includes several pathways involved in mitochondrial metabolism, which are diminished in AMPK knockout cells.

### Inhibition of AMPK sensitizes GB cells to nutrient starvation and hypoxia

**Fig. 2:**
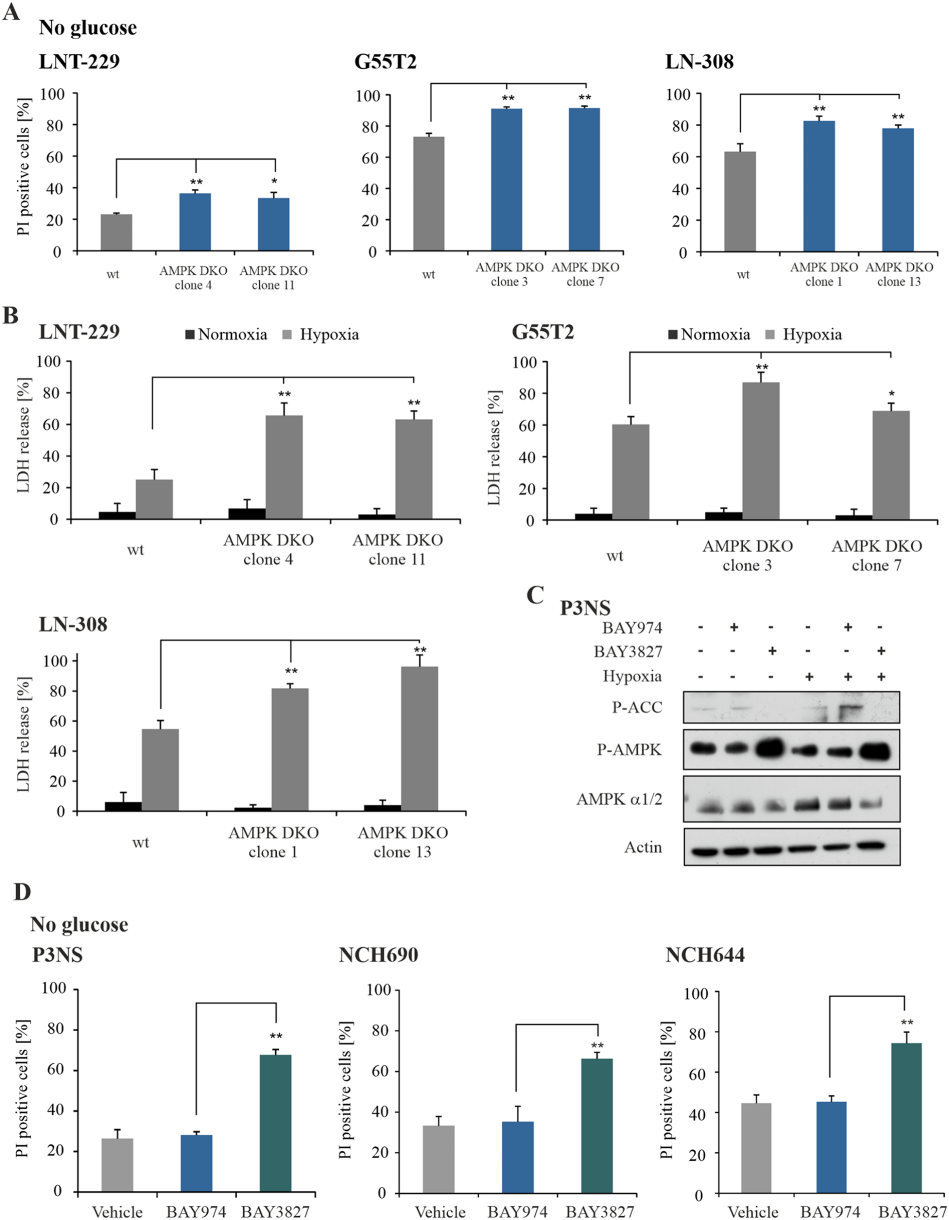
Inhibition of AMPK sensitizes GB cells to nutrient starvation and hypoxia (**A**) LNT-229, LN-308 and G55T2 wildtype (wt) and AMPK catalytic subunits double knockout (DKO) cells were treated in serum- and glucose-free DMEM for 24 h. Cell death was analyzed by PI staining and quantified by flow cytometry (*n* = 3, mean ± SD, **p < 0.01, Student’s t-test). (**B**) Cell death of LNT-229, LN-308 and G55T2 wildtype and AMPK DKO was analyzed by an LDH release assay after incubation of the cells in serum-free DMEM containing 2 mM glucose in normoxia or hypoxia (0.1% O_2_) (*n* = 4, mean ± SD, *p < 0.05, **p < 0.01, Student’s t-test). (**C**) Primary GB cells (P3NS) were treated with vehicle (DMSO), 1 µM BAY974 or 1 µM BAY3827 in serum-free medium containing 2 mM glucose for 8 h in normoxia or hypoxia (0.1% O_2_). Cellular lysates were analyzed by immunoblot with antibodies for P-ACC, P-AMPK, AMPKα1/2 and actin. (**D**) Human primary glioblastoma cells P3NS, NCH60 and NCH644 cells were treated with vehicle (DMSO), 1 µM BAY974 or 1 µM BAY3827 in serum-free medium without glucose. Cell death was analyzed by PI staining and flow cytometry (*n* = 3, mean ± SD, **p < 0.01, Student’s t-test)

Because mitochondrial metabolism is a major determinant of tumor cell assertiveness during metabolic stress, we aimed to investigate the effect of defective AMPK signaling in human GB cell lines under conditions resembling the tumor microenvironment. To this end, we exposed LNT-229, G55T2 and LN-308 AMPK DKO cells to glucose starvation and hypoxia. Cells cultured in glucose-free medium showed a significantly higher rate of cell death compared to corresponding wildtype cells (Fig. 2A). Under hypoxic conditions with reduced glucose availability, the hypersensitivity of LNT-229, G55T2 and LN-308 AMPK DKO cells was even more pronounced (Fig. 2B). Besides the genetic model, effects of pharmacological inhibition of AMPK were analyzed using the specific chemical probe BAY3827. This potent compound targets the α1 subunit of AMPK [40]. In LNT-229 cells, treatment with BAY3827 led to reduced P-ACC levels. In contrast, P-AMPK levels paradoxically increased after BAY3827 treatment (Supplementary Fig. 2A). Under glucose starvation conditions pharmacological AMPK inhibition with BAY3827 also increased cell death compared to vehicle treated cells or cells treated with the inactive, but chemically similar control probe BAY974 (Supplementary Fig. 2B). Moreover, LNT-229 cells treated with BAY3827 were sensitized to hypoxia-induced cell death (Supplementary Fig. 2C). To transfer these observations, P3NS primary GB cells were treated with BAY974 or BAY3827. Comparable to LNT-229 cells, P-ACC levels were reduced after pharmacological AMPK inhibition, whereas P-AMPK levels increased (Fig. 2C). Furthermore, we found that BAY3827 drastically increased cell death when primary GB cells were treated with BAY3827 under glucose-free conditions (Fig. 2D). These results indicate an important role of AMPK for the adaptation to conditions of the tumor microenvironment in human GB cells.

**Fig. 3:**
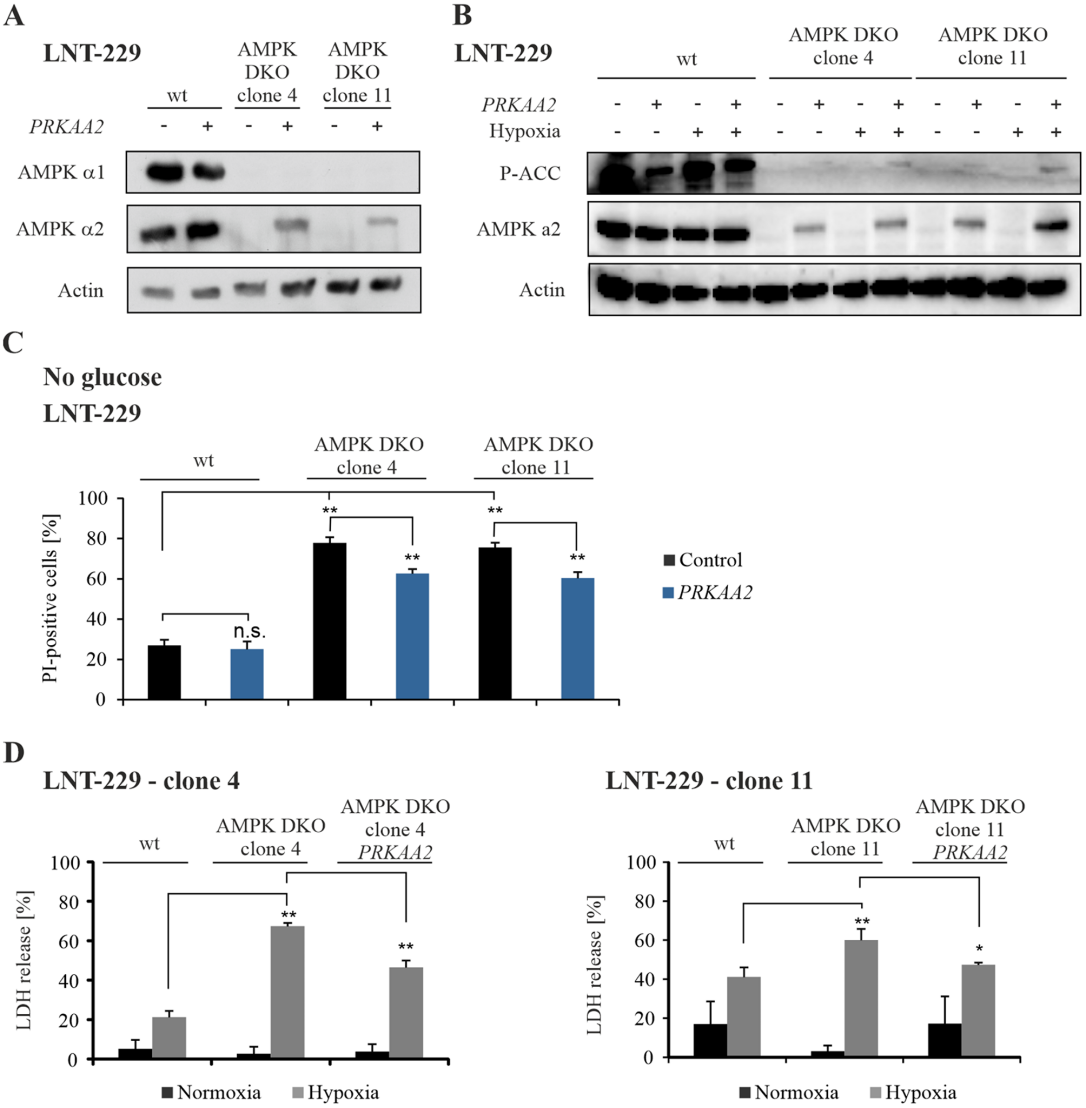
Retransfection of the catalytic subunit of AMPK in knockout cells restores adaptation to starvation conditions (**A**) LNT-229 wildtype (wt) and AMPK catalytic subunits double knockout (DKO) cells were stably transfected with empty vector (control) or *PRKAA2*. Cellular lysates were analyzed by immunoblot with antibodies for AMPK α1, AMPK α2 and actin. (**B**) Immunoblot analysis of LNT-229 wildtype and AMPK DKO PRKAA2 lysates treated in serum-free medium with 2 mM glucose for 8 h in normoxia or hypoxia (0.1% O_2_) was performed with antibodies for P-ACC, AMPK α2 and actin. (**C**) LNT-229 wildtype and AMPK DKO PRKAA2 cells were treated in serum-free medium without glucose. Cell death was analyzed by PI uptake and flow cytometry (*n* = 3, mean ± SD, n.s. not significant, **p < 0.01, Student’s t-test). (**D**) Cell death of LNT-229 wildtype and AMPK DKO cells was analyzed by LDH release assay after incubation in serum-free medium supplemented with 2 mM glucose in normoxia and hypoxia (0.1% O_2_) (*n* = 4, mean ± SD, *p < 0.05, **p < 0.01, Student’s t-test)

To confirm AMPK dependency of the observed effects under energy stress conditions, LNT-229 AMPK DKO cells were retransfected with *PRKAA2*, coding for the α2 subunit of AMPK (Fig. 3A). Cells reexpressing the α2 AMPK subunit showed a slight increase in P-ACC levels, which were however still reduced when compared to wildtype cells (Fig. 3B). Nevertheless, *PRKAA2* retransfected LNT-229 AMPK DKO cells showed reduced cell death under nutrient starvation conditions (Fig. 3C). Similarly, retransfected cells were partly protected from hypoxia-induced cell death compared to LNT-229 AMPK DKO cells (Fig. 3D).

### Mitochondrial abundance and activity are dependent on AMPK catalytic functionality

**Fig. 4:**
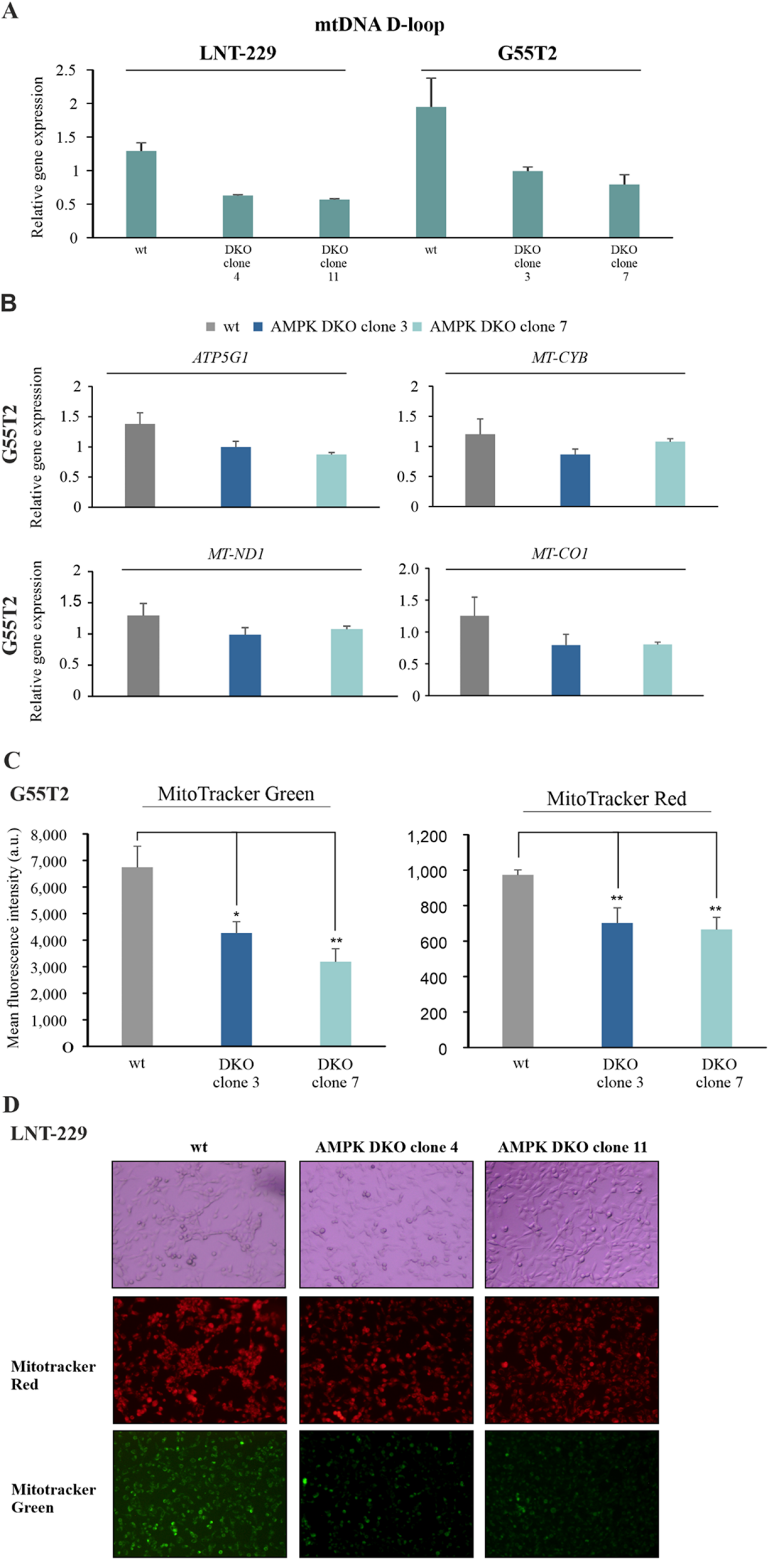
Mitochondrial mass and activity are impaired by AMPK catalytic subunits knockout in human GB cell lines (**A**) LNT-229 and G55T2 wildtype (wt) and AMPK catalytic subunits double knockout (DKO) cells were analyzed for the mRNA expression of *mtDNA D-loo*p by qPCR. *18 S* and *SDHA* were used for normalization (*n* = 3, mean ± SD). (**B**) mRNA expression of mitochondrial encoded as well as mitochondrial associated genes (*ATP5G1*, *MT-CYB*, *MT-ND1* and *MT-CO1*) of G55T2 wildtype and AMPK DKO cells was determined by qPCR. *18 S* and *SDHA* were used as housekeeping genes for normalization (*n* = 3, mean ± SD). (**C**) G55T2 wildtype and AMPK DKO cells were incubated in serum-free DMEM for 24 h. Cells were stained with 100 nM MitoTracker Green or 100 nM MitoTracker Red for 20 min and analyzed by flow cytometry. Mean fluorescence intensities are shown (*n* = 3, mean ± SD, *p < 0.05, **p < 0.01, Student’s t-test). (**D**) LNT-229 wildtype and AMPK DKO cells were treated as described in (**C**). Bright-field (upper panel) and fluorescence microscopy (RFP channel: middle panel, GFP channel: lower panel) were used for analysis (48x magnification). Representative images are shown

Previous studies have reported that AMPK regulates mitochondrial biogenesis and activity depending on the cellular energy status [13, 14, 41]. To investigate whether mitochondrial mass and biogenesis is regulated in an AMPK-dependent manner in human GB cells, LNT-229 and G55T2 wildtype and AMPK DKO cells were analyzed for mitochondrial DNA content by qPCR analysis with primers targeting the mtDNA D-loop. In both cell lines AMPK DKO resulted in a reduced mitochondrial DNA content compared to wildtype cells (Fig. 4A). We further analyzed mRNA expression levels of several mitochondrial encoded genes as well as mitochondria-associated genes in G55T2 wildtype and AMPK DKO cells (Fig. 4B). Mitochondrial encoded genes (*mtDNA D-loop*, *MT-CYB*, *MT-ND1* and *MT-CO1*) as well as the mitochondria associated gene *ATP5G1* were downregulated in AMPK DKO cells compared to wildtype cells. In line with this observation, mitochondrial abundance (Fig. 4C, left panel) and mitochondrial membrane potential (Fig. 4C, right panel) were reduced which was also confirmed microscopically in LNT-229 wildtype and AMPK DKO cells (Fig. 4D).

Pharmacological inhibition of oxidative phosphorylation was induced by IACS-010759, which selectively interferes with mitochondrial respiratory complex I [42–44]. IACS-010759 treatment of LNT-229 wildtype cells led to increased phosphorylation of AMPK and ACC under glucose deprived conditions in normoxia and hypoxia (Supplementary Fig. 3A). Under hypoxic conditions P-AMPK and P-ACC signal intensities were induced compared to normoxic conditions. Furthermore, oxygen consumption was reduced by IACS-010759 treatment in LNT-229 wildtype and AMPK DKO cells. Moreover, AMPK DKO cells showed reduced oxygen consumption compared to wildtype cells, which was more pronounced under oxidative phosphorylation inhibition (Supplementary Fig. 3B).

### AMPK catalytic subunits knockout cells are sensitized to 2-Deoxyglucose treatment

**Fig. 5:**
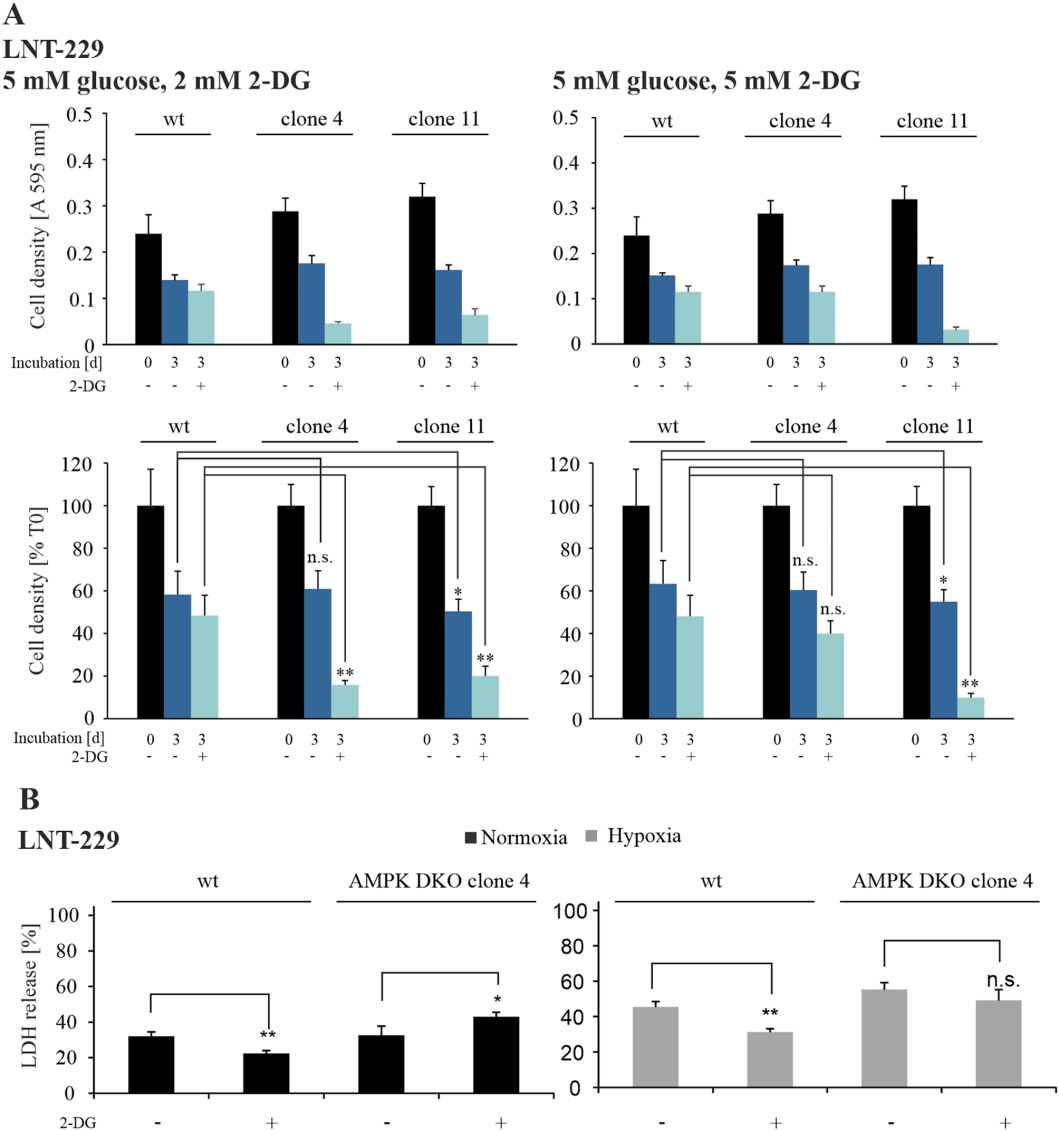
AMPK catalytic subunits double knockout cells are sensitized to 2-deoxyglucose treatment (**A**) CV staining of LNT-229 wildtype and AMPK catalytic subunits double knockout (DKO) cells was performed after treatment with vehicle (DMSO), 2 mM (left panel) or 5 mM (right panel) 2-DG in serum-free medium supplemented with 5 mM glucose for 72 h. Results are shown as absorption at 595 nm and relative to T0, reflecting the respective cell density at the beginning of the experiment. (**B**) LNT-229 wildtype and AMPK DKO cells were treated with vehicle (DMSO) or 2 mM 2-DG in serum-free DMEM with 2 mM glucose in normoxia or hypoxia (0.1% O_2_). LDH release assay was used for cell death analysis (*n* = 4, mean ± SD, n.s. not significant, *p < 0.05, **p < 0.01, Student’s t-test)

The glycolysis inhibitor 2-deoxyglucose (2-DG) impairs glucose metabolism by accumulation of the unconvertable intermediate 2-deoxyglucose-6-phosphate, which inhibits phosphoglucose isomerase [45]. We investigated AMPK-dependent effects of glycolysis inhibition by using AMPK DKO cells. Cell growth analyses showed that 2-DG treatment led to reduced cell densities in wildtype LNT-229 cells but this effect was more pronounced in AMPK DKO cells in both tested glucose/2-DG ratios (Fig. 5A). Under normoxic conditions 2-DG treatment resulted in protection from glucose-starvation induced cell death in wildtype, while the opposite effect was observed in AMPK DKO cells. 2-DG treatment under concomitant glucose and oxygen starvation led to protection from hypoxia-induced cell death in LNT-229 wildtype cells, whereas AMPK DKO cells did not benefit from 2-DG treatment (Fig. 5B).

### AMPK catalytic subunits knockout impairs tumor growth and leads to prolonged survival in an orthotopic mouse glioma model

The effects of AMPK DKO on tumor formation and progression were investigated in in vivo experiments. Chorioallantoic membrane (CAM) assays represent a simple in vivo approach to analyze biological effects of tumor formation and progression [46–48]. The G55T2 orthotopic model has been shown to cause necrotic tumor growth similar to human GB [49]. Under such conditions, AMPK-mediated effects are presumably more pronounced than in less aggressive tumor models. G55T2 wildtype and AMPK DKO cells were first allowed to grow on the CAM of fertilized chicken eggs (Supplementary Fig. 4A). After 8 days of incubation G55T2 wildtype cells formed significantly larger tumors with up to 70% increase in weight compared to G55T2 AMPK DKO cells (Supplementary Fig. 4B). IHC analysis of FFPE tissue of CAM tumors showed P-AMPK positive staining only in a small fraction of G55T2 AMPK DKO cells and no signal for P-ACC staining (Supplementary Fig. 4C). P-ACC expression was found to be dependent on tumor cell localization with lower expression in the outer and higher expression in the tumor center of G55T2 wildtype CAM tumors (Supplementary Fig. 4C). CA IX is known as surrogate marker for hypoxia and its expression is therefore increased in tumor regions with low oxygen supply [50, 51]. IHC staining showed that CA IX expression was induced in G55T2 wildtype CAM tumors, especially in the tumor center whereas G55T2 AMPK DKO CAM tumors showed only low expression of CA IX (Supplementary Fig. 4D).

**Fig. 6:**
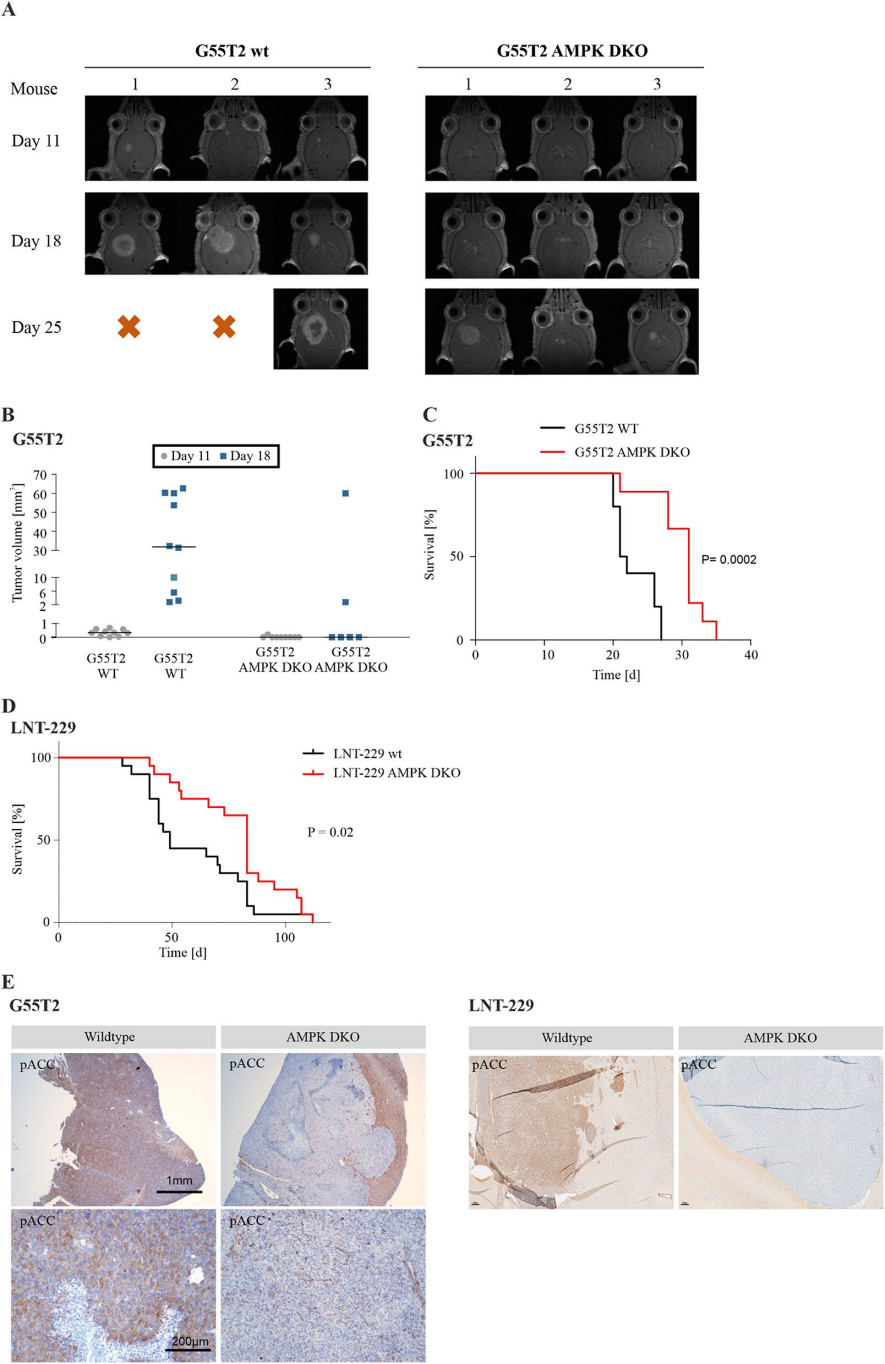
Knockout of AMPK catalytic subunits impairs tumor growth in vivo (**A**) Athymic nude mice were intracranially injected with 1 × 10^5^ G55T2 wildtype (wt) or AMPK catalytic subunits double knockout (DKO) cells (*n* = 10 mice per group). MRI measurements were performed on day 11, 18 and 25 after tumor cell injection. Images of 3 mice per group are shown exemplarily. (**B**) Tumor volumes were calculated based on MRI measurements on day 11 and 18 using the ITK Snap software. (**C**) Survival of mice (*n* = 10 per group) injected with G55T2 wildtype or AMPK DKO cells was analyzed by Kaplan-Meier Plot. Significance was tested using Log-Rank test. (**D**) Survival of mice (*n* = 20 per group) injected with LNT-229 wildtype or AMPK DKO cells was analyzed by Kaplan-Meier Plot. Significance was tested using Log-Rank test. Data of two independent experiments were pooled. (**E**) G55T2 wildtype and AMPK DKO tumors (left panel) were analyzed immunohistochemically with antibody for P-ACC. Scale bar represents 200 μm (10x magnification, bottom panel) or 1 mm (2x magnification, upper panel). LNT-229 wildtype and AMPK DKO tumors (right panel) were also analyzed with antibody for P-ACC

In addition to the CAM assays, tumor formation, growth and overall survival was analyzed in an orthotopic mouse model. Here, G55T2 AMPK DKO cells showed delayed tumor formation compared to G55T2 wildtype cells in MRI measurements (Fig. 6A). While all animals injected with G55T2 wildtype cells developed tumors that were detectable by MRI at day 18, this was only the case for two of nine mice injected with G55T2 AMPK DKO cells. In line with these results, volumetric analyses based on MRI measurements showed significantly larger tumors in mice injected with G55T2 wildtype cells at day 18 after tumor cell injection (Fig. 6B). With 31 versus 21.5 days in G55T2 AMPK DKO versus wildtype tumor bearing mice, median survival of mice with G55T2 AMPK DKO tumors was approximately 50% increased (Fig. 6C). Similarly, survival of mice with LNT-229 AMPK DKO tumors was prolonged (Fig. 6D) and inhibition of AMPK signaling in DKO tumors was confirmed via IHC staining for P-ACC (Fig. 6E).

## Discussion

Precise sensing of the cellular energy state is an important prerequisite to coordinate metabolism for sustained tumor growth in the GB microenvironment. AMPK orchestrates metabolism by increasing catabolism and inhibiting anabolism [11, 52, 53]. Accordingly, chronic AMPK activation has been reported in GB cells [18].

We provide evidence that functional AMPK is essential for adaptation of human GB cells to energy starvation with direct effects on GB cell survival (Supplementary Fig. 5). AMPK was induced under nutrient-deprived as well as hypoxic conditions (Fig. 1D), while double knockout of the catalytic subunits α1 and α2 sensitized human GB cells to cell death induced by glucose starvation and hypoxia, both mimicking characteristic conditions of the GB microenvironment (Fig. 2A, B, D). Previous studies had reported increased levels of the AMPK subunits α1, β1 and γ1 on mRNA level compared to normal brain tissue as well as increased levels of phosphorylated AMPK in GB cells as an indication of a potential relevance of AMPK in GB [18]. Gene suppression of the β1 regulatory AMPK subunit has been shown to result in downregulation of cellular bioenergetics and reduced tumor growth in vivo [18]. Nevertheless, in this model, phosphorylation of AMPK was not fully inhibited because of a sustained catalytic AMPK activity. Indeed, double knockout of the catalytic subunits α1 and α2 is necessary for a precise analysis of AMPK specific effects (Fig. 3B-D). Transfection of AMPK DKO cells with *PRKAA2*, encoding for the α2 subunit, partially rescued cell death under glucose starvation and hypoxia (Fig. 3C, D). Of note, transfection with PRKAA2 did not lead to protein levels comparable to the wildtype AMPK protein level and thus could explain the partial rescue of those cells (Fig. 3A).

We also found similar effects in primary GB cells by employing pharmacological AMPK inhibition with BAY3827 (Fig. 2C, D, Supplementary Fig. 2A-C). In contrast to the frequently used AMPK inhibitor Compound C, which is known to lack AMPK specificity, we found a good selectivity of BAY3827 [40]. In both, LNT-229 and primary GB cells (P3NS, NCH690 and NCH644), BAY3827 treatment increased cell death under glucose starvation and hypoxia as it was also observed in the AMPK DKO model (Fig. 2C-D, Supplementary Fig. 2A-C). Moreover, BAY3827 treatment did not influence hypoxia-induced cell death rates in AMPK DKO cells demonstrating an absence of off-target effects (Supplementary Fig. 2C). Selective pharmacological AMPK inhibition could be a new therapeutic option for GB treatment by sensitizing GB cells to local conditions of the tumor microenvironment.

AMPK modulates mitochondrial biogenesis and homeostasis dependent on the cellular energy state [41]. Only little is known about the role of AMPK for mitochondria in GB cells. In the β1 gene suppression model, primary GB cells were characterized by reduced mitochondrial activity and mass [18]. Here, we demonstrate that abolishing catalytic AMPK activity by α1/α2 double knockout induced a significant reduction of mitochondrial DNA content and mass (Fig. 4A, C, D - MitoTracker Green) together with reduced mitochondrial membrane potential indicating lower mitochondrial activity (Fig. 4C, D - MitoTracker Red). In line with this observation, mRNA levels of mitochondrial encoded and mitochondria associated genes decreased in AMPK DKO cells compared to wildtype GB cells (Fig. 4B). In this context, PGC-1α, encoded by *PPARGC1A*, a master regulator of mitochondrial biogenesis has been found to be AMPK-dependently activated *via* p38MAPK activation leading to an enhanced mitochondrial activity and biogenesis to improve ATP synthesis to overcome energy depletion under nutrient-deprived conditions [15]. Interestingly similar to AMPK knockout we have previously shown that gene suppression of PGC-1α resulted in prolonged survival in an orthotopic mouse model [54]. This indicates that (some) downstream effects of AMPK are potentially mediated by PGC-1α. The essential role of AMPK to sustain mitochondrial activity for ATP production under energy starvation conditions, was further confirmed by the fact that inhibition of oxidative phosphorylation and AMPK DKO both resulted in a comparable reduction of oxygen consumption in LNT-229 cells (Supplementary Fig. 3B). Taken together, deregulated mitochondrial capacity to produce ATP appears to be an important consequence of defective AMPK activity.

Aerobic glycolysis is known to be an important route to metabolize glucose in tumor cells [55, 56]. Therefore, tumor cells are more susceptible to the treatment with glycolysis inhibitors like 2-DG [57]. In consequence to glycolysis inhibition, ATP is depleted and AMPK is activated [8, 58]. Our results demonstrate that AMPK DKO cells show an impaired adaptation to energy starvation conditions and thus it seems plausible that these cells are more sensitive to glycolysis inhibition than wildtype cells. In line, LNT-229 AMPK DKO cells showed decreased cell growth under glucose-deprived conditions combined with 2-DG treatment compared to LNT-229 wildtype cells (Fig. 5A). Moreover, treatment of AMPK DKO cells with 2-DG led to increased cell death compared to LNT-229 wildtype cells, which were protected from glucose starvation-mediated cell death because of an increased AMPK activation resulting in the inhibition of catabolic processes and activation of ATP generation to restore cellular energy storage. AMPK inhibition together with glycolysis inhibition (e.g. with 2-DG) could therefore be beneficial to render GB cells more susceptible to conditions of the tumor microenvironment. With regard to treatment conditions, both temozolomide and radiotherapy induce DNA damage triggering ATP consuming DNA repair pathways [59–61]. Similarly, temozolomide-induced damage of mitochondrial DNA as well as radiotherapy induced generation of mitochondrial reactive oxygen species interfere with mitochondrial function [62, 63], which could also trigger energy shortage due to decreased ATP production in the mitochondria. Under such circumstances, tumor cells would most likely display an increased dependency on AMPK to sustain energy supply rendering them specifically sensitive to AMPK inhibition. Nevertheless, systemic AMPK inhibition, especially as part of a systemic combination therapy approach (e.g. with temozolomide or inhibitors of glycolysis), could induce relevant off-target toxicity and therefore careful dose-finding studies when designing in vivo experiments or phase 1 clinical trials would be mandated.

Findings of our in vivo models demonstrate the importance of AMPK for GB growth. In line with previous studies, where gene suppression of the regulatory β1 subunit was sufficient to confer prolonged survival in tumor-bearing mice, we found that knockout of the catalytic AMPK subunits led to impaired tumor formation of CAM tumors and in mouse experiments (Fig. 6A, B, Supplementary Fig. 4B). Moreover, survival of AMPK DKO tumor bearing mice was significantly prolonged (Fig. 6C, D) and IHC staining for P-ACC was reduced in AMPK DKO tumors (Fig. 6E, Supplementary Fig. 4C). In this context, AMPK could be essential for adaptation to conditions of the tumor microenvironment and exploring potential synergistic effects with glycolysis inhibition is an important future topic.

## Conclusions

In summary, adaptation to the tumor microenvironment is a central prerequisite for sustained tumor growth in solid cancers. Our study demonstrates that AMPK mediates adaptation of GB cells to energy stress conditions. Therefore, AMPK inhibition is a promising strategy for GB treatment either as monotherapy or to sensitize tumor cells to other metabolically active therapies.

## Electronic supplementary material

Below is the link to the electronic supplementary material.

Supplementary Material 1

## Data Availability

The mass spectrometry proteomics data have been deposited to the ProteomeXchange Consortium via the PRIDE54 partner repository with the dataset identifier PXD055976. All other datasets used in the current study are available from the corresponding author upon reasonable request.

## References

[1] Monteiro AR, Hill R, Pilkington GJ, Madureira PA. The role of hypoxia in glioblastoma invasion. Cells 2017; 6(4). 10.3390/cells6040045 PMC5755503 29165393

[2] Stupp R, Mason WP, van den Bent MJ, Weller M, Fisher B, Taphoorn MJB, et al. Radiotherapy plus concomitant and adjuvant Temozolomide for glioblastoma. N Engl J Med. 2005;352(10):987–96. 15758009 10.1056/NEJMoa043330

[3] Hanahan D, Weinberg RA. Hallmarks of cancer: the next generation. Cell. 2011;144(5):646–74. 21376230 10.1016/j.cell.2011.02.013

[4] Weyandt JD, Thompson CB, Giaccia AJ, Rathmell WK. Metabolic alterations in Cancer and their potential as therapeutic targets. Am Soc Clin Oncol Educ Book. 2017;37:825–32. 28561705 10.14694/EDBK_175561 PMC5954416

[5] Hardie DG, Schaffer BE, Brunet A. AMPK: an Energy-Sensing pathway with multiple inputs and outputs. Trends Cell Biol. 2016;26(3):190–201. 26616193 10.1016/j.tcb.2015.10.013 PMC5881568

[6] Lin S-C, Hardie DG. AMPK: sensing glucose as well as cellular energy status. Cell Metab. 2018;27(2):299–313. 29153408 10.1016/j.cmet.2017.10.009

[7] Zhang C-S, Hawley SA, Zong Y, Li M, Wang Z, Gray A, et al. Fructose-1,6-bisphosphate and aldolase mediate glucose sensing by AMPK. Nature. 2017;548(7665):112–6. 28723898 10.1038/nature23275 PMC5544942

[8] Garcia D, Shaw RJ. AMPK: mechanisms of cellular energy sensing and restoration of metabolic balance. Mol Cell. 2017;66(6):789–800. 28622524 10.1016/j.molcel.2017.05.032 PMC5553560

[9] Xiao B, Sanders MJ, Underwood E, Heath R, Mayer FV, Carmena D, et al. Structure of mammalian AMPK and its regulation by ADP. Nature. 2011;472(7342):230–3. 21399626 10.1038/nature09932 PMC3078618

[10] Hardie DG, Ross FA, Hawley SA. AMPK: a nutrient and energy sensor that maintains energy homeostasis. Nat Rev Mol Cell Biol. 2012;13(4):251–62. 22436748 10.1038/nrm3311 PMC5726489

[11] Hardie DG, Pan DA. Regulation of fatty acid synthesis and oxidation by the AMP-activated protein kinase. Biochem Soc Trans. 2002;30(Pt 6):1064–70. 12440973 10.1042/bst0301064

[12] Laplante M, Sabatini DM. mTOR signaling in growth control and disease. Cell. 2012;149(2):274–93. 22500797 10.1016/j.cell.2012.03.017 PMC3331679

[13] Herzig S, Shaw RJ. AMPK: guardian of metabolism and mitochondrial homeostasis. Nat Rev Mol Cell Biol. 2018;19(2):121–35. 28974774 10.1038/nrm.2017.95 PMC5780224

[14] Zhang C-S, Lin S-C. AMPK promotes autophagy by facilitating mitochondrial fission. Cell Metab. 2016;23(3):399–401. 26959181 10.1016/j.cmet.2016.02.017

[15] Chaube B, Malvi P, Singh SV, Mohammad N, Viollet B, Bhat MK. AMPK maintains energy homeostasis and survival in cancer cells via regulating p38/PGC-1α-mediated mitochondrial biogenesis. Cell Death Discov. 2015;1:15063. 27551487 10.1038/cddiscovery.2015.63 PMC4979508

[16] Faubert B, Vincent EE, Poffenberger MC, Jones RG. The AMP-activated protein kinase (AMPK) and cancer: many faces of a metabolic regulator. Cancer Lett. 2015;356(2 Pt A):165–70. 24486219 10.1016/j.canlet.2014.01.018

[17] Jiang H, Liu W, Zhan S-K, Pan Y-X, Bian L-G, Sun B, et al. GSK621 targets glioma cells via activating AMP-Activated protein kinase signalings. PLoS ONE. 2016;11(8):e0161017. 27532105 10.1371/journal.pone.0161017 PMC4988667

[18] Chhipa RR, Fan Q, Anderson J, Muraleedharan R, Huang Y, Ciraolo G, et al. AMP kinase promotes glioblastoma bioenergetics and tumour growth. Nat Cell Biol. 2018;20(7):823–35. 29915361 10.1038/s41556-018-0126-z PMC6113057

[19] Shackelford DB, Abt E, Gerken L, Vasquez DS, Seki A, Leblanc M, et al. LKB1 inactivation dictates therapeutic response of non-small cell lung cancer to the metabolism drug phenformin. Cancer Cell. 2013;23(2):143–58. 23352126 10.1016/j.ccr.2012.12.008 PMC3579627

[20] Bain J, Plater L, Elliott M, Shpiro N, Hastie CJ, McLauchlan H, et al. The selectivity of protein kinase inhibitors: a further update. Biochem J. 2007;408(3):297–315. 17850214 10.1042/BJ20070797 PMC2267365

[21] Dasgupta B, Seibel W. Compound C/Dorsomorphin: its use and misuse as an AMPK inhibitor. Methods Mol Biol. 2018;1732:195–202. 29480476 10.1007/978-1-4939-7598-3_12

[22] Liu X, Chhipa RR, Nakano I, Dasgupta B. The AMPK inhibitor compound C is a potent AMPK-independent antiglioma agent. Mol Cancer Ther. 2014;13(3):596–605. 24419061 10.1158/1535-7163.MCT-13-0579 PMC3954437

[23] Ronellenfitsch MW, Brucker DP, Burger MC, Wolking S, Tritschler F, Rieger J, et al. Antagonism of the mammalian target of Rapamycin selectively mediates metabolic effects of epidermal growth factor receptor Inhibition and protects human malignant glioma cells from hypoxia-induced cell death. Brain. 2009;132(Pt 6):1509–22. 19416948 10.1093/brain/awp093

[24] Eckerich C, Schulte A, Martens T, Zapf S, Westphal M, Lamszus K. RON receptor tyrosine kinase in human gliomas: expression, function, and identification of a novel soluble splice variant. J Neurochem. 2009;109(4):969–80. 19519771 10.1111/j.1471-4159.2009.06027.x

[25] Wischhusen J, Naumann U, Ohgaki H, Rastinejad F, Weller M. CP-31398, a novel p53-stabilizing agent, induces p53-dependent and p53-independent glioma cell death. Oncogene. 2003;22(51):8233–45. 14614447 10.1038/sj.onc.1207198

[26] Lorenz NI, Sittig ACM, Urban H, Luger A-L, Engel AL, Münch C, et al. Activating transcription factor 4 mediates adaptation of human glioblastoma cells to hypoxia and Temozolomide. Sci Rep. 2021;11(1):14161. 34239013 10.1038/s41598-021-93663-1 PMC8266821

[27] SGC Frankfurt. Available from: URL: http://www.thesgc.org/click-trust

[28] Golebiewska A, Hau A-C, Oudin A, Stieber D, Yabo YA, Baus V, et al. Patient-derived organoids and orthotopic xenografts of primary and recurrent gliomas represent relevant patient avatars for precision oncology. Acta Neuropathol. 2020;140(6):919–49. 33009951 10.1007/s00401-020-02226-7 PMC7666297

[29] Wanka C, Brucker DP, Bähr O, Ronellenfitsch M, Weller M, Steinbach JP, et al. Synthesis of cytochrome C oxidase 2: a p53-dependent metabolic regulator that promotes respiratory function and protects glioma and colon cancer cells from hypoxia-induced cell death. Oncogene. 2012;31(33):3764–76. 22120717 10.1038/onc.2011.530

[30] Grady JE, Lummis WL, Smith CG. An improved tissue culture assay. III. Alternate methods for measuring cell growth. Cancer Res. 1960;20:1114–7. 13828726

[31] Steinbach JP, Eisenmann C, Klumpp A, Weller M. Co-inhibition of epidermal growth factor receptor and type 1 insulin-like growth factor receptor synergistically sensitizes human malignant glioma cells to CD95L-induced apoptosis. Biochem Biophys Res Commun. 2004;321(3):524–30. 15358139 10.1016/j.bbrc.2004.06.175

[32] Thiepold A-L, Lorenz NI, Foltyn M, Engel AL, Divé I, Urban H, et al. Mammalian target of Rapamycin complex 1 activation sensitizes human glioma cells to hypoxia-induced cell death. Brain. 2017;140(10):2623–38. 28969371 10.1093/brain/awx196

[33] Gerstmeier J, Possmayer A-L, Bozkurt S, Hoffmann ME, Dikic I, Herold-Mende C, et al. Calcitriol promotes differentiation of glioma Stem-Like cells and increases their susceptibility to Temozolomide. Cancers. 2021;13(14):3577. 34298790 10.3390/cancers13143577 PMC8303292

[34] Langfelder P, Horvath S. WGCNA: an R package for weighted correlation network analysis. BMC Bioinformatics. 2008;9:559. 19114008 10.1186/1471-2105-9-559 PMC2631488

[35] Morabito S, Reese F, Rahimzadeh N, Miyoshi E, Swarup V. HdWGCNA identifies co-expression networks in high-dimensional transcriptomics data. Cell Rep Methods. 2023;3(6):100498. 37426759 10.1016/j.crmeth.2023.100498 PMC10326379

[36] Xu S, Hu E, Cai Y, Xie Z, Luo X, Zhan L, et al. Using clusterprofiler to characterize multiomics data. Nat Protoc. 2024;19(11):3292–320. 39019974 10.1038/s41596-024-01020-z

[37] Yushkevich PA, Piven J, Hazlett HC, Smith RG, Ho S, Gee JC, et al. User-guided 3D active contour segmentation of anatomical structures: significantly improved efficiency and reliability. NeuroImage. 2006;31(3):1116–28. 16545965 10.1016/j.neuroimage.2006.01.015

[38] Ruiz-Moreno C, Salas SM, Samuelsson E, Brandner S, Kranendonk ME, Nilsson M, et al. Harmonized single-cell landscape. intercellular crosstalk and tumor architecture of glioblastoma; 2022.

[39] Ravi VM, Will P, Kueckelhaus J, Sun N, Joseph K, Salié H, et al. Spatially resolved multi-omics Deciphers bidirectional tumor-host interdependence in glioblastoma. Cancer Cell. 2022;40(6):639–e65513. 35700707 10.1016/j.ccell.2022.05.009

[40] Lemos C, Schulze VK, Baumgart SJ, Nevedomskaya E, Heinrich T, Lefranc J, et al. The potent AMPK inhibitor BAY-3827 shows strong efficacy in androgen-dependent prostate cancer models. Cell Oncol (Dordr). 2021;44(3):581–94. 33492659 10.1007/s13402-020-00584-8 PMC12980684

[41] Toyama EQ, Herzig S, Courchet J, Lewis TL, Losón OC, Hellberg K, et al. Metabolism. AMP-activated protein kinase mediates mitochondrial fission in response to energy stress. Science. 2016;351(6270):275–81. 26816379 10.1126/science.aab4138 PMC4852862

[42] Tsuji A, Akao T, Masuya T, Murai M, Miyoshi H. IACS-010759, a potent inhibitor of glycolysis-deficient hypoxic tumor cells, inhibits mitochondrial respiratory complex I through a unique mechanism. J Biol Chem. 2020;295(21):7481–91. 32295842 10.1074/jbc.RA120.013366 PMC7247293

[43] Molina JR, Sun Y, Protopopova M, Gera S, Bandi M, Bristow C, et al. An inhibitor of oxidative phosphorylation exploits cancer vulnerability. Nat Med. 2018;24(7):1036–46. 29892070 10.1038/s41591-018-0052-4

[44] Vangapandu HV, Alston B, Morse J, Ayres ML, Wierda WG, Keating MJ, et al. Biological and metabolic effects of IACS-010759, an oxphos inhibitor, on chronic lymphocytic leukemia cells. Oncotarget. 2018;9(38):24980–91. 29861847 10.18632/oncotarget.25166 PMC5982765

[45] Pajak B, Siwiak E, Sołtyka M, Priebe A, Zieliński R, Fokt I et al. 2-Deoxy-d-Glucose and its analogs: from diagnostic to therapeutic agents. Int J Mol Sci 2019; 21(1). 10.3390/ijms21010234 PMC6982256 31905745

[46] Lokman NA, Elder ASF, Ricciardelli C, Oehler MK. Chick Chorioallantoic membrane (CAM) assay as an in vivo model to study the effect of newly identified molecules on ovarian cancer invasion and metastasis. Int J Mol Sci. 2012;13(8):9959–70. 22949841 10.3390/ijms13089959 PMC3431839

[47] Richardson M, Singh G. Observations on the use of the avian Chorioallantoic membrane (CAM) model in investigations into angiogenesis. Curr Drug Targets Cardiovasc Haematol Disord. 2003;3(2):155–85. 12769641 10.2174/1568006033481492

[48] Deryugina EI, Quigley JP. Chick embryo Chorioallantoic membrane model systems to study and visualize human tumor cell metastasis. Histochem Cell Biol. 2008;130(6):1119–30. 19005674 10.1007/s00418-008-0536-2 PMC2699943

[49] Seidel S, Garvalov BK, Wirta V, von Stechow L, Schänzer A, Meletis K, et al. A hypoxic niche regulates glioblastoma stem cells through hypoxia inducible factor 2 alpha. Brain. 2010;133(Pt 4):983–95. 20375133 10.1093/brain/awq042

[50] Vaupel P, Mayer A. Hypoxia in cancer: significance and impact on clinical outcome. Cancer Metastasis Rev. 2007;26(2):225–39. 17440684 10.1007/s10555-007-9055-1

[51] McLendon R. Carbonic anhydrase IX as a marker of hypoxia in gliomas: A narrative review. Glioma. 2020;3(3):97.

[52] Jeon S-M, Chandel NS, Hay N. AMPK regulates NADPH homeostasis to promote tumour cell survival during energy stress. Nature. 2012;485(7400):661–5. 22660331 10.1038/nature11066 PMC3607316

[53] Kim J, Kundu M, Viollet B, Guan K-L. AMPK and mTOR regulate autophagy through direct phosphorylation of Ulk1. Nat Cell Biol. 2011;13(2):132–41. 21258367 10.1038/ncb2152 PMC3987946

[54] Bruns I, Sauer B, Burger MC, Eriksson J, Hofmann U, Braun Y, et al. Disruption of peroxisome proliferator-activated receptor γ coactivator (PGC)-1α reverts key features of the neoplastic phenotype of glioma cells. J Biol Chem. 2019;294(9):3037–50. 30578297 10.1074/jbc.RA118.006993 PMC6398126

[55] WARBURG O. On respiratory impairment in cancer cells. Science. 1956;124(3215):269–70. 13351639

[56] Vander Heiden MG, Cantley LC, Thompson CB. Understanding the Warburg effect: the metabolic requirements of cell proliferation. Science. 2009;324(5930):1029–33. 19460998 10.1126/science.1160809 PMC2849637

[57] Aft RL, Zhang FW, Gius D. Evaluation of 2-deoxy-D-glucose as a chemotherapeutic agent: mechanism of cell death. Br J Cancer. 2002;87(7):805–12. 12232767 10.1038/sj.bjc.6600547 PMC2364258

[58] Barialai L, Strecker MI, Luger A-L, Jäger M, Bruns I, Sittig ACM, et al. AMPK activation protects astrocytes from hypoxia–induced cell death. Int J Mol Med. 2020;45(5):1385–96. 32323755 10.3892/ijmm.2020.4528 PMC7138264

[59] Kaina B, Christmann M, Naumann S, Roos WP. MGMT: key node in the battle against genotoxicity, carcinogenicity and apoptosis induced by alkylating agents. DNA Repair (Amst). 2007;6(8):1079–99. 17485253 10.1016/j.dnarep.2007.03.008

[60] Cucchi D, Gibson A, Martin SA. The emerging relationship between metabolism and DNA repair. Cell Cycle. 2021;20(10):943–59. 33874857 10.1080/15384101.2021.1912889 PMC8172156

[61] Morgan MA, Lawrence TS. Molecular pathways: overcoming radiation resistance by targeting DNA damage response pathways. Clin Cancer Res. 2015;21(13):2898–904. 26133775 10.1158/1078-0432.CCR-13-3229 PMC4494107

[62] Averbeck D, Rodriguez-Lafrasse C. Role of mitochondria in radiation responses: epigenetic, metabolic, and signaling impacts. Int J Mol Sci 2021; 22(20). 10.3390/ijms222011047 PMC8541263 34681703

[63] Oliva CR, Nozell SE, Diers A, McClugage SG, Sarkaria JN, Markert JM, et al. Acquisition of Temozolomide chemoresistance in gliomas leads to remodeling of mitochondrial electron transport chain. J Biol Chem. 2010;285(51):39759–67. 20870728 10.1074/jbc.M110.147504 PMC3000957

