# Supplementary material for: AMP-activated protein kinase mediates adaptation of glioblastoma cells to conditions of the tumor microenvironment
Source: J Exp Clin Cancer Res. 2025 Mar 24;44:104. doi: 10.1186/s13046-025-03346-2 (PMC11931870; doi:10.1186/s13046-025-03346-2)
Supplement: Supplementary file 1 — Supplementary Material 1 [file 13046_2025_3346_MOESM1_ESM.docx]

**Supplementary Information**

**AMP-kinase mediates adaptation of glioblastoma cells to conditions of the tumor microenvironment**

Nadja I. Lorenz et al.

**Supplementary Figures**


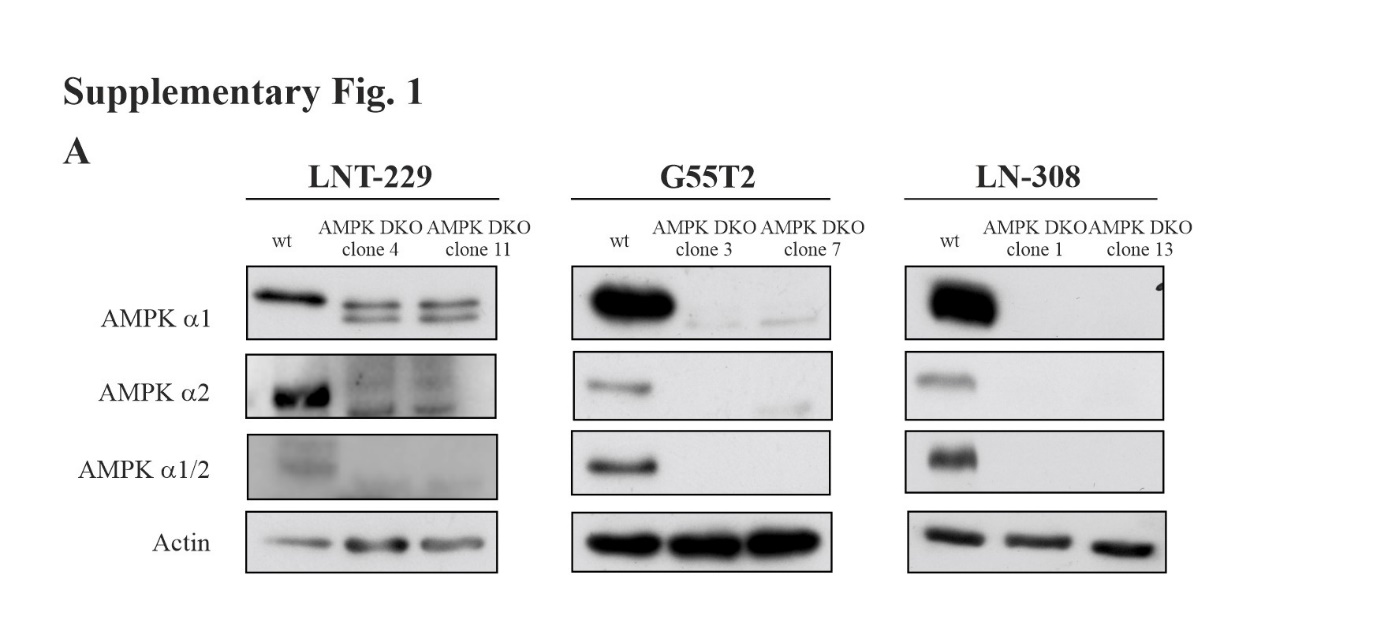


**Supplementary Fig. 1: AMPK catalytic subunits double knockout prevents downstream signaling**

(A) Cellular lysates of LNT-229, G55T2 and LN-308 wildtype (wt) and AMPK catalytic subunits double knockout (DKO) cells were analyzed by immunoblot with antibodies for AMPKα1, AMPKα2, AMPKα1/2 and actin.


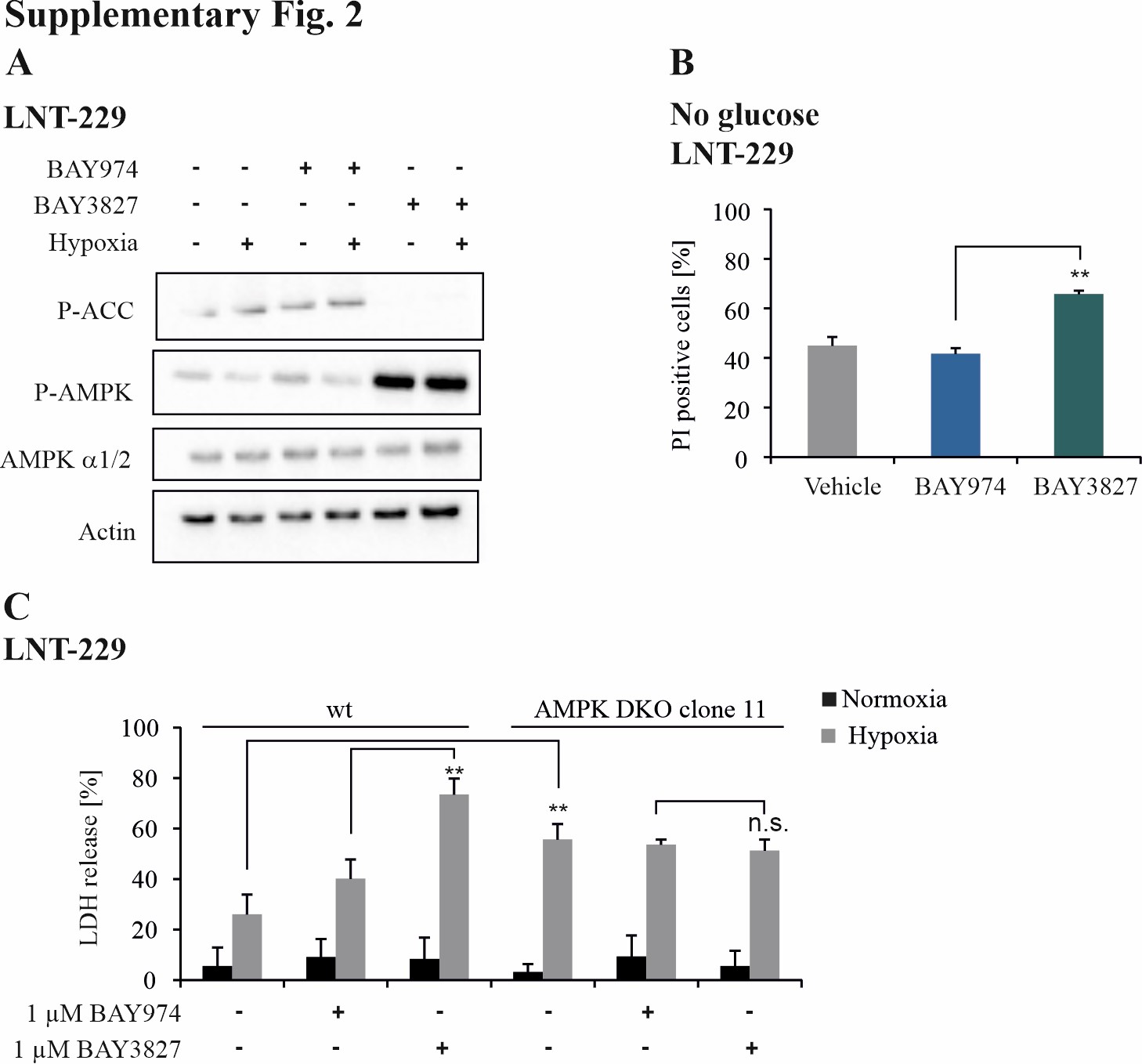


**Supplementary Fig. 2: Pharmacological AMPK inhibition sensitizes GB cells to glucose starvation- and hypoxia-induced cell death.**

(A) LNT-229 cells were treated with vehicle, 1 µM BAY974 (negative control) or 1 µM BAY3827 in serum-free DMEM containing 2 mM glucose in normoxia or hypoxia (0.1 % O_2_) for 8 h as indicated. Immunoblot analysis was performed with antibodies for P-ACC, P-AMPK, AMPK and actin. (B) LNT-229 cells were treated with vehicle (DMSO), 1 µM BAY974 or 1 µM BAY3827 in glucose-free medium as indicated. PI staining was used for cell death analysis and quantified by FACS measurement (n=3, mean ± SD, **p<0.01, Student’s t-test). (C) LNT‑229 wildtype and AMPK DKO cells were incubated in serum-free medium containing 2 mM glucose and vehicle (DMSO), 1 µM BAY974 or 1 µM BAY3927 under normoxic or hypoxic (0.1 % O_2_) conditions as indicated. Cell death was analyzed by LDH release assay (n=4, mean ± SD, n.s. not significant, **p<0.01, Student’s t-test).

**
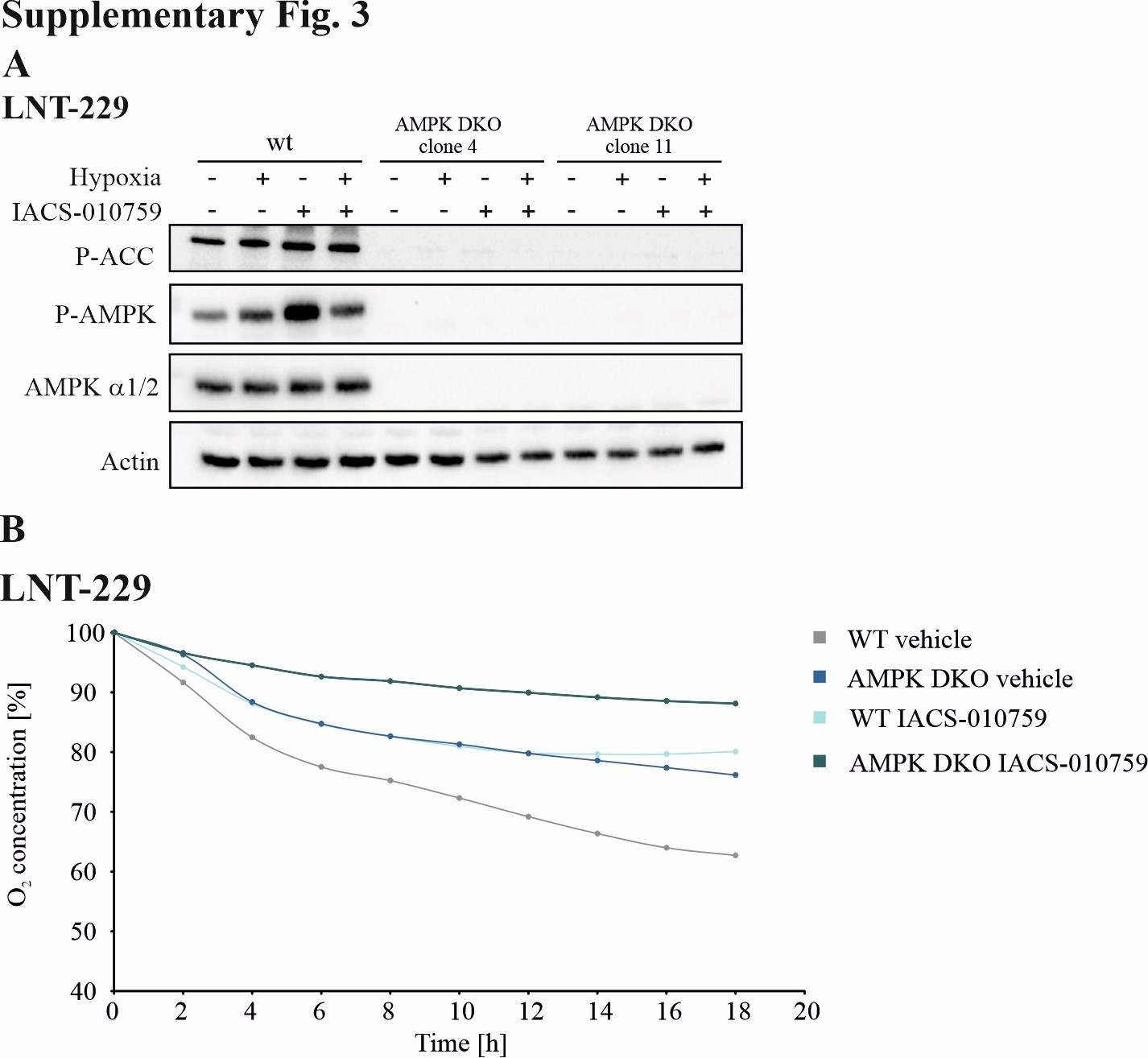
**

**Supplementary Fig. 3: Inhibition of oxidative phosphorylation induces AMPK activation in human GB cells.**

(A) LNT-229 wildtype and AMPK DKO cells were treated with vehicle (DMSO) or 100 nM IACS-010759 in serum-free medium containing 2 mM glucose in normoxia or hypoxia (0.1% O_2_) for 8 h. Immunoblot analysis was performed using antibodies for P-ACC, P-AMPK, AMPK α1/2 and actin. (B) LNT-229 cells were treated with vehicle (DMSO) or 100 nM IACS-010759 in serum-free DMEM without glucose restriction. Oxygen consumption was measured by a fluorescence-based assay.

**
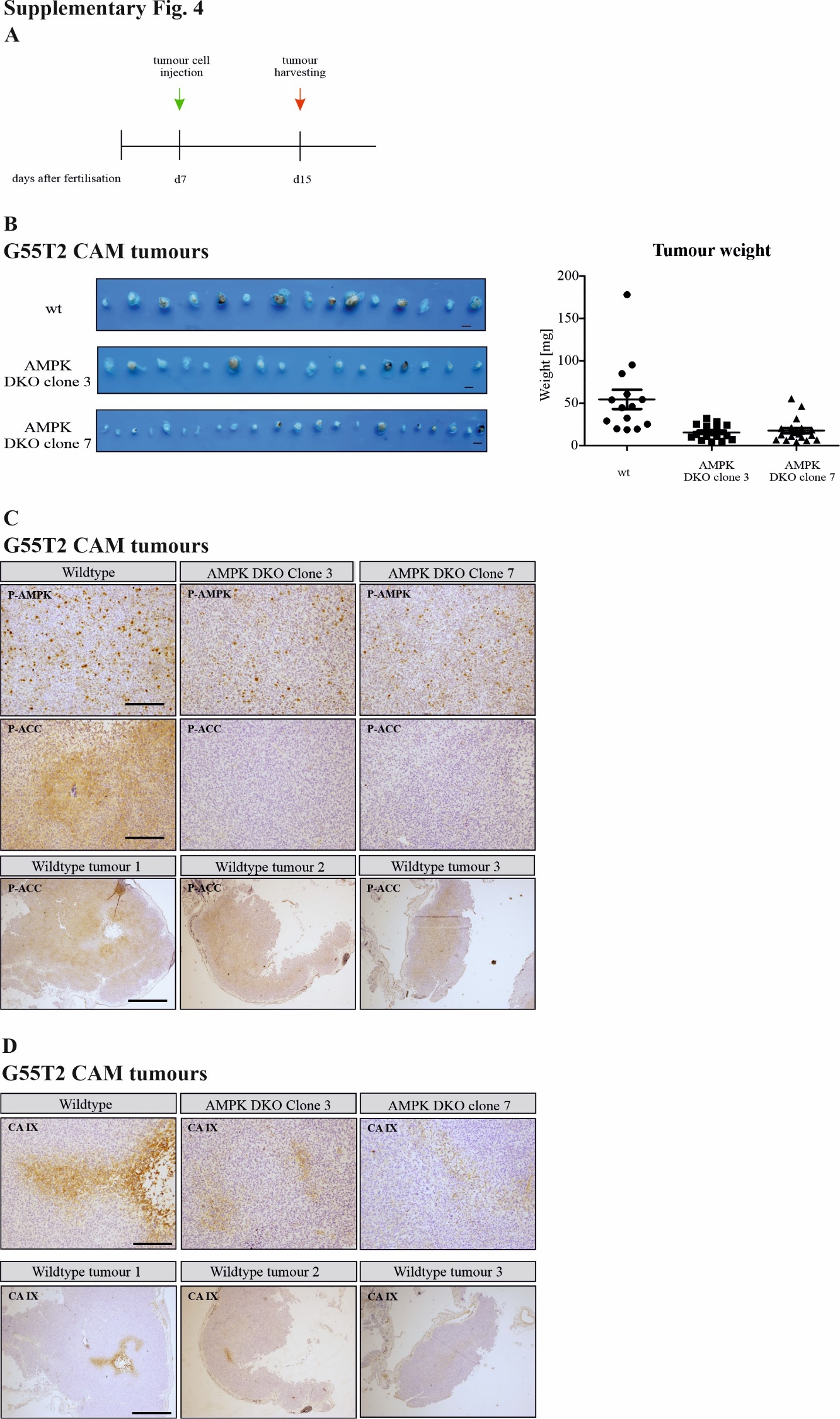
**

**Supplementary Fig. 4: AMPK catalytic subunits double knockout (DKO) impairs tumor growth and metabolic adaptation in a CAM model.**

(A, B) Chicken eggs were injected with 2 x 10^6^ G55T2 wildtype (wt) or AMPK catalytic subunits double knockout (DKO) cells on day 7 post fertilization and incubated for an additional 8 days. Tumors were isolated on day 15, weight was measured and tumors were fixed for immunohistochemical analysis. Images of isolated tumors are shown (scale bar: 5 mm). (C) G55T2 wildtype and AMPK DKO CAM tumors were analyzed immunohistochemically with antibodies for P-AMPK and P-ACC. Scale bar represents 200 µm (10x magnification, upper and middle panel) or 1 mm (2x magnification, bottom panel). (D) Isolated CAM tumors were stained for CA IX. Scale bar represents 200 µm (upper panel), 1 mm (2x magnification, bottom panel).

**
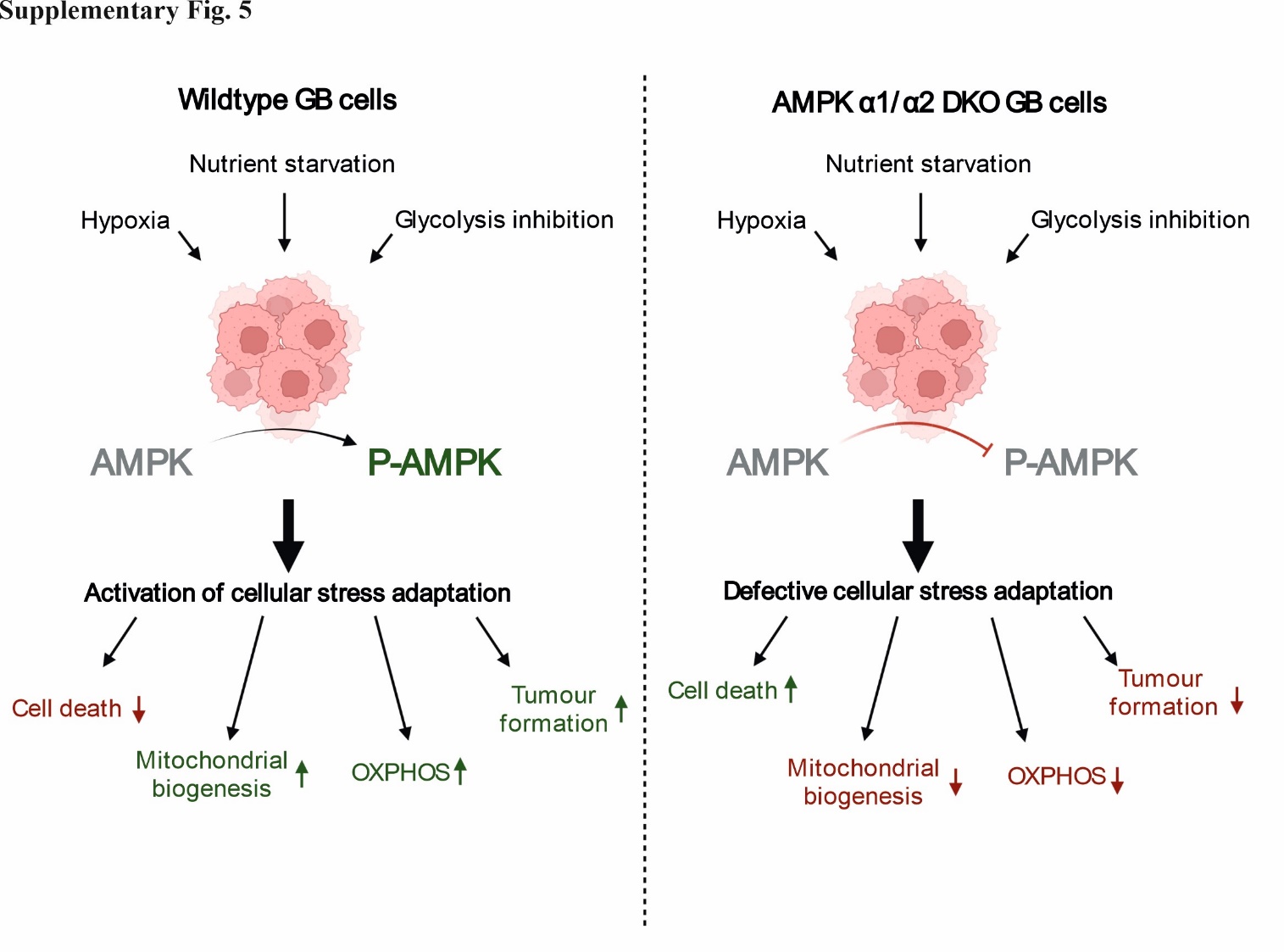
**

**Supplementary Fig. 5: AMPK catalytic subunits double knockout sensitizes GB cells to conditions of the tumor microenvironment.**

GB cells are challenged by conditions of the tumor microenvironment with low oxygen and nutrient availability. Activation of AMPK enables metabolic adaptation including catabolic processes (OXPHOS) and mitochondrial biogenesis to promote tumor cell survival and growth (left panel). Double knockout of catalytic subunits α1 and α2 impairs AMPK signaling causing a defective response to energy starvation with in consequence impaired tumor formation (right panel). Figure created with biorender.com.

**Supplementary Table 1**

| MSigDB v2023.2.Hs gene set | Genes |
| --- | --- |
| AMP-activated protein kinase | *RRAGD*, *STRADB*, *RRAGB*, *PPM1A*, *CAB39L*, *TSC2*, *RHEB*, *PRKAG2*, *LAMTOR3*, *PRKAB1*, *LAMTOR2*, *STK11*, *PRKAB2*, *PRKAA1*, *LAMTOR5*, *CAB39*, *RPTOR*, *LAMTOR1*, *RRAGA*, *PRKAA2*, *TSC1*, *MLST8*, *SLC38A9*, *PRKAG1*, *LAMTOR4*, *MTOR*, *STRADA* |

**Supplementary Methods**

**Mass Spectrometry sample preparation and liquid chromatography MS**

**Sample preparation for mass spectrometry**

Lysate precipitation was carried out by adding 3 volumes of ice-cold methanol, 1 volume of chloroform, and 2.5 volumes of water. Following centrifugation at 14,000g for 15 minutes at 4°C, the upper aqueous phase was removed, and 3 volumes of ice-cold methanol were introduced. The samples were mixed, and the proteins were pelleted by centrifuging at 14,000g for 5 minutes at 4°C. The supernatant was discarded, and the pellets were washed once more with ice-cold methanol. Protein pellets were dried at room temperature for subsequent use. Proteins were then resuspended in 8 M Urea/10 mM EPPS pH 8.2. Protein concentration was measured using the Bradford assay, and 100 µg of protein per sample was digested by diluting to 1 M Urea with 10 mM EPPS pH 8.2 and incubating overnight at 37°C with LysC (Wako Chemicals, Neuss, Germany) at a 1:50 (w/w) ratio and Trypsin (Promega, Madison, WI, USA, V5113) at a 1:100 (w/w) ratio. The digests were acidified with TFA (0.2% vol/vol), and tryptic peptides were purified using tC18 SepPak (50 mg, Waters WAT054955, Milford, MA, USA). Peptide concentrations were determined with a μBCA assay (ThermoFisher Scientific, 23235), and 10 µg of peptides per sample were labeled with TMT reagents (Thermo Scientific, 90406). TMT-labeled samples were normalized to equimolar ratios for each channel after a single injection measurement by LC-MS/MS. The labeled peptide samples were then pooled and fractionated.

**High pH micro-flow fractionation**

Peptide fractionation was conducted using high-pH liquid chromatography on a micro-flow HPLC system (Dionex U3000 RSLC, Thermo Scientific). For this process, 45 µg of pooled and purified TMT-labeled peptides, which had been resuspended in Solvent A (5 mM ammonium bicarbonate, 5% ACN), were loaded onto a C18 column (XSelect CSH, 1 mm x 150 mm, 3.5 µm particle size; Waters). The peptides were separated via a multistep gradient ranging from 3% to 60% Solvent B (5 mM ammonium bicarbonate, 90% ACN) over a 65-minute period, with a flow rate of 30 µl/min. Peptides were collected every 43 seconds from minute 2 to minute 69, resulting in 96 fractions that were subsequently combined into 24 fractions. The combined fractions were then dried using a vacuum concentrator and reconstituted in 2% ACN, 0.1% TFA for subsequent LC-MS analysis.

**Liquid chromatography mass spectrometry (LC-MS^3^)**

All mass spectrometry data were collected in centroid mode using an Orbitrap Fusion Lumos mass spectrometer connected to an easy-nLC 1200 nano HPLC system with a nanoFlex ion source (ThermoFisher Scientific, Waltham, MA, USA). Peptide separation was performed on a custom-made 22 cm long, 75 µm ID fused-silica column, packed in-house with 1.9 µm C18 particles (ReproSil-Pur, Dr. Maisch, Ammerbuch-Entringen, Germany) and kept at 50°C using an integrated column oven (Sonation, Biberach, Germany). The HPLC solvents consisted of 0.1% formic acid in water (Buffer A) and 0.1% formic acid, 80% acetonitrile in water (Buffer B). Approximately 400 ng of each fraction was loaded onto the column and eluted over a 90-minute linear gradient from 10% to 40% Buffer B.

For proteome analysis, a synchronous precursor selection (SPS) multi-notch MS3 method was used to minimize ratio compression as previously described. Full scan MS spectra (350-1400 m/z) were acquired at a resolution of 120,000 at m/z 200, with a maximum injection time of 100 ms and an AGC target value of 4 x 10^5. The most intense precursors with charge states between 2 and 6 from each full scan were chosen for fragmentation using the “Top Speed” mode with a cycle time of 1.5 seconds and isolated with a quadrupole isolation window of 0.7 Th. MS2 scans were performed in the ion trap (Turbo) with a maximum injection time of 50 ms, an AGC target value of 1.5 x 10^4, and fragmented using CID with a normalized collision energy (NCE) of 35%. For quantification, SPS-MS3 scans were carried out on the 10 most intense MS2 fragment ions, with an isolation window of 0.7 Th (MS) and 2 m/z (MS2). These ions were fragmented using HCD with an NCE of 65% (TMTclassic) and analyzed in the Orbitrap at a resolution of 50,000 at m/z 200, a scan range of 100-500 m/z, an AGC target value of 1.5 x 10^5, and a maximum injection time of 86 ms. To avoid repeated sequencing of previously acquired precursors, a dynamic exclusion of 60 seconds and 7 ppm was applied, and advanced peak determination was disabled. All spectra were acquired in centroid mode.

**Proteomics data analysis**

The acquired raw data was analyzed with Proteome Discoverer 2.4 (ThermoFisher Scientific). The SequenceHT node was used for database searches of MS2 spectra. Protein identifications were made using the human trypsin-digested proteome (Homo sapiens SwissProt database, TaxID: 9606, version March 12, 2020). Contaminants were identified using the MaxQuant “contamination.fasta” file for quality control. Fixed modifications included TMT (+229.163) at the N-terminus and lysine (K), as well as carbamidomethylation (C, +57.021) at cysteine residues. Dynamic modifications were set for methionine oxidation (M, +15.995) and acetylation (+42.011) at the protein N-terminus. The precursor mass tolerance was set to 10 ppm and fragment mass tolerance to 0.6 Da. Default percolator settings in Proteome Discoverer were applied to filter peptide spectrum matches (PSMs) at a 0.01 false discovery rate. Reporter ion quantification was conducted with default settings in the consensus workflow. The protein file from Proteome Discoverer was then exported to .csv format for further processing. Normalized abundances from the protein file, after removal of contaminants and completely empty values, were used for statistical analysis. Differential expression analysis was performed using the limma package within NormalyzerDE in R Studio.^2^. Gene annotation for protein accessions were collated using artMS package^3^.

**References**

1. McAlister GC, Nusinow DP, Jedrychowski MP, et al. MultiNotch MS3 enables accurate, sensitive, and multiplexed detection of differential expression across cancer cell line proteomes. *Anal Chem*. 2014;86(14):7150-7158. doi:10.1021/ac502040v

2. Willforss J, Chawade A, Levander F. NormalyzerDE: Online Tool for Improved Normalization of Omics Expression Data and High-Sensitivity Differential Expression Analysis. *Journal of Proteome Research*. 2019;18(2):732-740. doi:10.1021/acs.jproteome.8b00523

3. *ArtMS.* Bioconductor; 2018. https://bioconductor.org/packages/artMS.
